# Moderate selenium mitigates hand grip strength impairment associated with elevated blood cadmium and lead levels in middle-aged and elderly individuals: insights from NHANES 2011–2014

**DOI:** 10.3389/fphar.2023.1324583

**Published:** 2023-12-14

**Authors:** Yafeng Liang, Junqi Wang, Tianyi Wang, Hangyu Li, Chaohui Yin, Jialin Liu, Yulong Wei, Junxing Fan, Shixing Feng, Shuangqing Zhai

**Affiliations:** ^1^ Beijing University of Chinese Medicine, Beijing, China; ^2^ Dongzhimen Hospital, Beijing University of Chinese Medicine, Beijing, China; ^3^ School of Management, Beijing University of Chinese Medicine, Beijing, China; ^4^ School of Life and Science, Beijing University of Chinese Medicine, Beijing, China; ^5^ School of Resources and Environment, Henan Agricultural University, Zhengzhou, Henan, China; ^6^ Dongfang Hospital, Beijing University of Chinese Medicine, Beijing, China; ^7^ School of Acupuncture-Moxibustion and Tuina, Beijing University of Chinese Medicine, Beijing, China; ^8^ Henan Provincial Health Talent Center, Zhengzhou, Henan, China; ^9^ Centre France Chine de la Médecine Chinoise, Selles sur Cher, France; ^10^ School of Traditional Chinese Medicine, Beijing University of Chinese Medicine, Beijing, China

**Keywords:** heavy metals, toxicity, selenium, cadmium, lead, hand grip strength

## Abstract

**Background:** Selenium (Se) has been reported to have an antagonistic effect on heavy metals in animals. Nevertheless, there is a lack of epidemiological research examining whether Se can mitigate the adverse effects of cadmium (Cd) and lead (Pb) on hand grip strength (HGS) in middle-aged and elderly individuals.

**Methods:** This study used data from the 2011–2014 National Health and Nutrition Examination Survey (NHANES). HGS measurements were conducted by trained examiners with a dynamometer. Concentrations of Se, Cd, and Pb in blood were determined *via* inductively coupled plasma mass spectrometry. We employed linear regression, restricted cubic splines, and quantile g-computation (qgcomp) to assess individual and combined associations between heavy metals and HGS. The study also explored the potential influence of Se on these associations.

**Results:** In both individual metal and multi-metal models adjusted for confounders, general linear regression showed Se’s positive association with HGS, while Cd and Pb inversely related to it. At varying Se-Cd and Se-Pb concentrations, high Se relative to low Se can attenuate Cd and Pb’s HGS impact. An inverted U-shaped correlation exists between Se and both maximum and combined HGS, with Se’s benefit plateauing beyond approximately 200 μg/L. Stratified analysis by Se quartiles reveals Cd and Pb’s adverse HGS effects diminishing as Se levels increase. Qgcomp regression analysis detected Se alleviating HGS damage from combined Cd and Pb exposure. Subsequent subgroup analyses identified the sensitivity of women, the elderly, and those at risk of diabetes to HGS impairment caused by heavy metals, with moderate Se supplementation beneficial in mitigating this effect. In the population at risk for diabetes, the protective role of Se against heavy metal toxicity-induced HGS reduction is inhibited, suggesting that diabetic individuals should particularly avoid heavy metal-induced handgrip impairment.

**Conclusion:** Blood Cd and Pb levels are negatively correlated with HGS. Se can mitigate this negative impact, but its effectiveness plateaus beyond 200 μg/L. Women, the elderly, and those at risk of diabetes are more vulnerable to HGS damage from heavy metals. While Se supplementation can help, its protective effect is limited in high diabetes risk groups.

## 1 Introduction

Selenium (Se) is an essential trace element present in various dietary sources, including grains, nuts, vegetables, and different animal products (Fan et al., 2023). It holds pivotal roles in numerous health aspects, including its antioxidant properties (Li et al., 2017), bolstering the immune system (Razaghi et al., 2021), tumor prevention (Razaghi et al., 2021), maintaining cardiovascular health (Mohammadifard et al., 2019), and enhancing renal function (Wang et al., 2022). Yet, the balance of Se intake is crucial; while deficiency can be harmful, excessive intake can also pose health risks. Supplementation of Se has been linked with enhanced immune responses, particularly during pregnancy, autoimmune thyroid disorders, and post-severe illnesses (Schomburg, 2021). On the flip side, high Se levels might impair neurological functions and increase the risk of type 2 diabetes (Genchi et al., 2023). Intriguingly, a Mendelian randomized study suggested that high Se levels might be detrimental to renal function (Fu et al., 2022). However, in contrast, Se supplementation has shown promise in improving kidney function in individuals with acute kidney injury or chronic kidney diseases (Iglesias et al., 2013). This underscores a dose-response relationship concerning Se’s health effects. Furthermore, a study highlighted that combined supplementation of zinc (Zn) and Se could boost physical functionality in overweight individuals undergoing calorie-restricted diets, hinting at Se‘s potential benefits in enhancing physical performance (Zavros et al., 2023).

Heavy metals, in contrast to trace elements, pose health hazards regardless of their concentration. Persistent exposure to these toxic elements entails significant health challenges. Abundant research delineates that such exposures can catalyze a gamut of health complications, from cardiovascular ailments and cancers to diabetes and renal dysfunctions, etc (Yao et al., 2021). Two commonly encountered heavy metal contaminants, lead (Pb) and cadmium (Cd), amass in human systems owing to the biomagnification phenomenon in the food chain (Li et al., 2023). Pb exerts profound detrimental effects on the brain, injuring its vascular endothelium, leading to cerebral microvessel dysfunction. This can cause aberrations in cerebral blood circulation, enhancing the likelihood of intracranial carotid artery atherosclerosis, which in turn, raises the potential for cardiovascular complications or strokes (Lin et al., 2018). An assessment centered on elderly individuals unveiled a negative correlation between Cd exposure and Hand grip strength (HGS) (García-Esquinas and Rodríguez-Artalejo, 2017). In a parallel vein, research deduced that amplified Pb exposure adversely influenced grip strength, alluding to a probable linkage between pediatric HGS and heavy metal pollutants (Wu et al., 2022). Animal experiments indicate that the toxic effects of Pb on muscles primarily manifest in disrupting normal glucose metabolism processes, altering the activity of key enzymes, and adjusting energy metabolism, which may also damage muscle tissue structure. These physiological changes could negatively impact muscle function and quality, thereby adversely affecting overall health (Das et al., 2020). In another animal study, it was found that Pb exposure’s harm to skeletal muscle function mainly includes reduced muscle mass, decreased exercise capacity, weakened muscle strength, disrupted lipid metabolism, increased inflammatory responses, and upregulated expression of pro-apoptotic genes. These damages are likely due to lead’s direct effects on muscle cell structure, metabolism, and inflammatory pathways (He et al., 2023). Further research also shows that Cd’s damage to skeletal muscles mainly manifests as reduced muscle weight and strength, decreased exercise capability, disrupted lipid metabolism, intensified inflammatory responses, and enhanced expression of apoptotic genes. These injuries might originate from Cd’s direct toxic action on muscle cells and the associated inflammatory reactions (He et al., 2023). On the other hand, considering that the critical values for grip strength diagnostic criteria differ by gender, and the physiological differences between men and women, the impact of heavy metals on male and female grip strength may exhibit significant gender differences. However, research on the gender differences in the effects of heavy metals on male and female grip strength remains relatively insufficient. Existing literature suggests that men and women differ in risk factors associated with reduced grip strength (Sternäng et al., 2015). Studies have found that early childhood Pb exposure is associated with loss of brain structural volume, and this Pb exposure-related brain volume reduction is more pronounced in male participants (Cecil et al., 2008). Research on African Americans reveals significant lifetime Pb content differences, with women potentially facing higher health risks due to Pb release from bones (Theppeang et al., 2008). Additionally, the potential impact of Cd exposure on cardiovascular health indicators shows a statistically significant association in Caucasian men and Mexican-American women (Egwuogu et al., 2009). Given these ramifications, tackling the consequences of heavy metal exposure emerges as a paramount public health imperative.

HGS gauges an individual’s ability to grasp objects, reflecting the force exerted by hand muscles. A prevalent tool in clinical environments, HGS serves as an indicator of hand strength and overall muscle functionality (Wang et al., 2018). From a physiological standpoint, HGS typically peaks in early adulthood, maintains stability through midlife, and then gradually declines, predominantly impacting those in middle age and beyond (Dodds et al., 2014). Numerous clinical and epidemiological studies highlight HGS’s predictive value, associating it with both short-term and long-term health outcomes (Sayer and Kirkwood, 2015). In clinical contexts, a decreased HGS can signal potential post-operative complications, prolonged hospital stays, increased rates of hospital readmission, and general physical decline. Among the elderly, reduced HGS often corresponds with a loss of independence (Labott et al., 2019). Furthermore, epidemiological data suggest that even in healthy adults, a declining HGS can foretell forthcoming functional limitations, disabilities in old age, and an elevated risk of mortality (Norman et al., 2011). Given these implications, bolstering HGS stands out as a vital health strategy, especially for the middle-aged and elderly populations.

In essence, while moderate intake of Se offers health benefits, toxic heavy metals adversely impact HGS. This presents a critical query: Can Se counterbalance the detrimental effects of heavy metals, potentially safeguarding HGS by tempering their toxicity? Several experimental studies suggest that Se element could mitigate the negative impacts of heavy metals on animal cognition (Ren et al., 2022). A population-based study inferred that moderate Se intake might counter the lung function deterioration induced by Pb and Cd in humans (Fan et al., 2023). Another research centered on the elderly in China proposed that Se might neutralize the harmful effects of Pb and Cd on cognitive functions (Cheng et al., 2023). Consequently, it is conceivable that an optimal dose of Se can potentially counteract the damaging effects of heavy metal exposure. However, to translate this hypothesis into actionable clinical recommendations, it needs more expansive epidemiological research and rigorous clinical trials.

Given the existing research landscape, there’s a noticeable gap in understanding whether Se can counteract the decline in HGS due to heavy metals, especially Pb and Cd. Addressing this void, our study utilized data from the National Health and Nutrition Examination Survey (NHANES) to meticulously explore the relationships between blood concentrations of Se, Cd, and Pb and their collective impact on HGS. The insights from this study aim to broaden our understanding of how optimal Se intake might counterbalance the adverse effects of Cd and Pb on HGS. This knowledge could inform suitable Se supplementation recommendations and pave the way for pioneering therapeutic approaches to counteract HGS decline attributed to heavy metal toxicity.

## 2 Methods

### 2.1 Study population

In this study, we utilized data from the NHANES, which is designed to gather data emblematic of the civilian, non-institutionalized US population, aiming to discern the prevalence of significant diseases and pinpoint their associated risk factors. The datasets employed for this research were extracted from two NHANES survey cycles: 2011–2012 and 2013–2014. In our cross-sectional analysis, certain participants were omitted based on the following criteria: 1) individuals aged below 45, accounting for 13,524 participants; 2) those with absent data on Se, Pb, and Cd, totaling 2,047 participants; and 3) participants lacking handgrip test data, comprising 518 individuals.

Following these exclusions, this analysis encompassed 3,842 participants, primarily from the middle-aged and elderly demographics.

### 2.2 Assessment of HGS

The muscle strength/grip test component evaluated isometric HGS using a handgrip dynamometer. The grip test was conducted in a standing position unless the participant had physical limitations. This study measured participants’ muscle strength in two ways: maximum HGS and combined HGS. Participants performed the HGS test three times for both left and right hands. The study took the highest value out of the three tests as the participant’s maximum HGS; the combined HGS refers to the sum of the largest reading from each hand.

### 2.3 Assessment of Se, Cd, and Pb

Three elements of toxicological and nutritional interest including Cd, Pb, and Se in whole human blood specimens are processed, stored, and later shipped to the National Center for Environmental Health and the Centers for Disease Control and Prevention for test, which were measured directly using mass spectrometry after a simple dilution sample preparation step. Optimal amount of specimen is 1–2 mL. Request a minimum volume of 0.4 mL. Volume for one analytical measurement is 0.1 mL. No fasting or special diets are required before collection of blood. The lower detection limits for Pb were 0.25 μg/dL, 0.16 μg/L for Cd and 30 ug/L for Se, respectively. It is imperative to mention that values falling beneath the established LLODs for Pb, Cd, and Se are computed as half the LLOD value squared. All participants have signed the informed consent. More details about the whole blood metals can be seen in Supplementary Material (1.4 Supplementary Explanation on the Detailed Measurement Process of Se, Cd, and Lead).

### 2.4 Covariates

For this analysis, participants’ sociodemographic details, lifestyle attributes, and additional body metrics were considered. The sociodemographic data primarily captured age, sex, race (categorized into Mexican American, non-Hispanic white, non-Hispanic African American, other Hispanic, and other races), marital status (distinguished as married or unmarried), and educational level (delineated as having a bachelor’s degree or higher, or below). Lifestyle parameters encompassed aspects like alcohol intake (quantified by consuming a minimum of 12 alcoholic beverages annually), smoking habits (with serum cotinine levels serving as a nicotine exposure marker to evaluate smoke exposure), and body mass index (BMI). Other pertinent body metrics integrated into the study were systolic blood pressure (SBP), diastolic blood pressure (DBP), HbA1c levels, total cholesterol (TC), the metabolic equivalent of task (MET)score and HEI-2015 index. The specific details of covariates can be found in Supplementary Table SA3.

### 2.5 Statistical analysis

In this study, continuous variables are presented as mean ± SD, while categorical variables are described by their frequencies and percentages. Blood concentrations of heavy metals are represented by the median with the inter-quartile range (IQR). Depending on the data type, group characteristics were compared using the chi-square test, ANOVA, or the Kruskal–Wallis H-test. To investigate the relationship between exposure and HGS, we employed both univariate and multivariate linear regression models. These included an unadjusted model, a fully adjusted version, and a multi-metal model designed to account for confounders due to heavy metals. We further examined the effect of Se on the association between blood levels of heavy metals (Cd and Pb) and HGS by using multivariable linear regressions and restricted cubic splines based on blood Se quartiles. Additionally, the study explored the associations between four specific Se-Cd or Se-Pb exposure patterns and HGS. The quantile g-computation model was utilized to precisely determine the relationships between mixed heavy metal exposures and HGS. All statistical analyses were performed with R software (version 4.3.1). A two-tailed *p*-value of less than 0.05 was considered statistically significant. In the subgroup analysis, this study categorized participants into gender subgroups (male and female) and age subgroups (<60, ≥60). The results of the subgroup analysis are presented in Supplementary Tables S7-S8. In the sensitivity analysis, this study conducted additional adjustments for two variables that could potentially influence HGS levels, namely, MET score and HEI-2015. Detailed calculation methods and descriptions can be found in Supplementary Materials Section 1.1 and Section 1.2.

## 3 Results

### 3.1 Characteristics of selected population

Table 1 provides a detailed overview of the primary attributes of both the general population and the study’s participants, segmented according to Se quartiles from the NHANES dataset spanning 2011–2014. The cohort included 3,842 participants, displaying a near-even gender distribution: 48.9% male and 51.1% female, with an average age of 61.78 ± 10.65 years. Notably, individuals in the higher Se quartiles exhibited distinct features: they were generally younger, predominantly male, had a higher representation of non-Hispanic African Americans, exhibited greater academic achievements, were more frequently married, and presented a larger portion with lower BMI values. In contrast, participants with lower Se concentrations were more inclined towards smoking and alcohol consumption and had elevated blood pressure, TC, and HbA1c levels. A comprehensive overview of baseline attributes related to Se, Pb, and Cd can be found in Supplementary Table S1.

**TABLE 1:** Characteristics in participants stratified by the blood Se quartile.

Characteristics	Overall (n = 3,842)	Q1 (n = 961)	Q2 (n = 960)	Q3 (n = 960)	Q4 (n = 961)	*p-value*
Age, (year)	61.78 ± 10.65	63.16 ± 11.23	61.71 ± 10.65	61.2 ± 10.33	60.9 ± 10.24	<0.001
Gender, n (%)						<0.001
Female, n (%)	1964 ± 51.1	524 ± 54.5	533 ± 55.5	477 ± 49.7	430 ± 44.7	
Male, n (%)	1860 (48.9)	437 (45.5)	427 (44.5)	483 (50.3)	531 (55.3)	
Race, n (%)						<0.001
Mexican American	344 (9.0)	69 (7.2)	96 (10.0)	93 (9.7)	86 (8.9)	
Non-Hispanic white	383 (10.0)	110 (11.4)	89 (9.3)	103 (10.7)	81 (8.4)	
Non-Hispanic African American	1652 (43.0)	416 (43.3)	412 (42.9)	395 (41.1)	429 (44.)6	
Other Hispanic	965 (25.1)	283 (29.4)	246 (25.6)	241 (25.1)	195 (20.)3	
Other races	498 (13.0)	83 (8.6)	117 (12.2)	128 (13.3)	170 (17.)7	
College or above, n (%)	1998 ± 52.0	424 ± 44.2	486 ± 50.6	539 ± 56.2	549 ± 57.1	<0.001
Married, n (%)	2,252 ± 58.6	478 ± 49.7	562 ± 58.6	608 ± 63.3	604 ± 62.9	<0.001
Drinking, n (%)	2,559 ± 70.9	598 ± 67.5	628 ± 69.9	673 ± 73.1	660 ± 72.9	0.025
Cotinine, (ng/mL)	55.2.4 ± 135.24	71.57 ± 141.20	54.06 ± 124.94	43.24 ± 121.39	52.16 ± 149.89	<0.001
SBP, (mmHg)	128.87 ± 18.56	129.88 ± 20.51	128.95 ± 18.86	128.39 ± 17.75	128.27 ± 16.92	0.255
DBP, (mmHg)	70.63 ± 13.45	69.29 ± 13.82	70.68 ± 13.10	71.10 ± 13.97	71.45 ± 12.81	0.005
TC, (mg/dL)	196.22 ± 43.52	186.74 ± 40.61	195.03 ± 41.81	199.64 ± 44.20	203.33 ± 45.53	<0.001
BMI, (kg/m^2^)	29.31 ± 6.73	29.28 ± 7.38	29.52 ± 6.74	29.37 ± 6.58	29.06 ± 6.18	0.492
HbA1c, (%)	6.00 ± 1.16	5.93 ± 0.99	5.94 ± 1.08	6.00 ± 1.11	6.13 ± 1.39	<0.001
Max HGS, (Kg)	32.8 ± 10.33	30.9 ± 10.21	32.5 ± 10.36	33.6 ± 10.22	34.2 ± 10.22	<0.001
Combined HGS, (Kg)	65.6 ± 20.69	61.6 ± 20.35	65.0 ± 20.61	67.3 ± 20.66	68.4 ± 20.50	<0.001

**Note**: Percentages were computed including those with missing values. Data are expressed as numbers (percentages) for categorical variables and as w mean (standard deviations) for continuous variables; Q1: Se < 178 μg/L; Q2: 178–192 μg/L; Q3: 192–208 μg/L; Q4: Se ≥ 208 μg/L.

Abbreviation: BMI, body mass index; SBP, systolic blood pressure; DBP, diastolic blood pressure; TC, total cholesterol; HbA1c, hemoglobin A1c; HGS, hand grip strength.

The study’s results emphasize a significant positive correlation between maximum grip strength and Se concentrations. As Se levels increased, there was a marked improvement in maximum grip strength. This correlation was evident across quartiles, with measurements as follows: Quartile 1 averaged 30.9 ± 10.21 kg, Quartile 2 averaged 32.5 ± 10.36 kg, Quartile 3 averaged 33.6 ± 10.22 kg, and Quartile 4 averaged 34.2 ± 10.22 kg (*p* < 0.001). Similarly, the combined grip strength demonstrated a consistent trend across quartiles: Quartile 1 at 61.6 ± 20.35 kg, Quartile 2 at 65.0 ± 20.61 kg, Quartile 3 at 67.3 ± 20.66 kg, and Quartile 4 at 68.4 ± 20.50 kg (*p* < 0.001).

**FIGURE 1:**
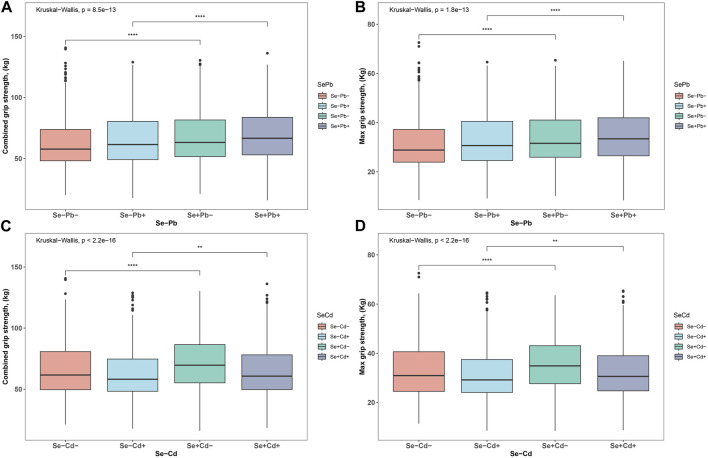
The distributions of HGS. **(A)** the distribution of Max HGS in the four patterns of Se-Pb exposure **(B)** the distribution of combined HGS in the four patterns of Se-Pb exposure **(C)** the distribution of Max HGS in the four patterns of Se-Cd exposure **(D)** the distribution of combined HGS in the four patterns of Se-Cd exposure. ***p < 0.001, **p < 0.01, *p < 0.05.

Figure 1 provides a comprehensive distribution analysis of both maximum and combined HGS across two primary exposure combinations: Cd-Se and Pb-Se. When assessing maximum HGS, the groups with Se presence, regardless of Cd or Pb exposure, consistently showcased superior HGS outcomes compared to their counterparts without Se (all *p* < 0.001). Specifically, the Se + Cd-group notably outperformed the Se- Cd-group, and similar patterns were observed for the Pb exposure groups. Turning attention to combined HGS, the presence of Se again emerged as a beneficial factor, enhancing grip strength across both Cd and Pb exposures. Notably, the Se + Cd-group demonstrated a heightened combined HGS over the Se- Cd-group, with similar superior results observed for the Se + groups in Pb exposure scenarios (all *p* < 0.001). Collectively, these findings underscore the potential protective role of Se in counteracting the detrimental impacts of both Cd and Pb on HGS.

### 3.2 Association of individual metals with HGS

Table 2 summarizes the results from a general linear regression analysis examining the associations between Cd, Pb, and Se levels with HGS. In the unadjusted single-metal model, Cd was negatively correlated with HGS, while both Pb and Se demonstrated positive correlations with HGS. In the adjusted single-metal model, Cd retained its negative association with HGS, and Se continued to correlate positively. However, the relationship between Pb and HGS became negative. In the adjusted multi-metal model, the negative association of Cd with HGS persisted (maximum: β = −0.45, CI [-0.87, −0.04]; combined: β = −1.10, CI [-1.94, −0.26]). Pb exhibited a negative relationship with HGS (maximum: β = −0.20, CI [-0.33, −0.08]; combined: β = −0.35, CI [-0.61, −0.11]), while the positive association of Se with HGS remained consistent (maximum: β = 0.01, CI [0.00, 0.02]; combined: β = 0.02, CI [0.00, 0.03]).

**TABLE 2:** Associations between Cd, Pb, Se and HGS using general linear regression.

Outcomes	Single metal (unadjusted)		Single metal (adjusted)^a^		Multiple metals (adjusted)^b^	
	β (95% CI)	*p-value*	β (95% CI)	*p-value*	β (95% CI)	*p-value*
Max HGS						
Cd	−0.71 (−1.26, −0.18)	0.008	−0.56 (−0.98, −0.15)	0.008	−0.45 (−0.87, −0.04)	0.033
Pb	0.44 (0.27, 0.63)	<0.001	−0.02 (−0.35, −0.10)	<0.001	−0.20 (−0.33, −0.08)	0.002
Se	0.03 (0.02, 0.04)	<0.001	0.01 (0.00, 0.02)	0.006	0.01 (0.00, 0.02)	0.010
Combined HGS						
Cd	−1.58 (-2.65, −0.50)	0.004	−1.29 (-2.13, −0.46)	0.002	−1.10 (−1.94, −0.26)	0.006
Pb	0.89 (0.53, 1.26)	<0.001	−0.41 (-0.66, −0.16)	0.001	−0.35 (−0.61, −0.11)	0.010
Se	0.07 (0.04, 0.09)	<0.001	0.02 (0.00, 0.03)	<0.001	0.02 (0.00, 0.03)	0.011

^a^ Covariates in adjusted single-metal models included age, gender, race, education levels, marital status, cotinine, drinking status, BMI, SBP, DBP, HbA1c, TC.

^b^ Covariates in multiple-metal models included covariates in adjusted single-metal models and the other two metals except for the independent variable.

**FIGURE 2:**
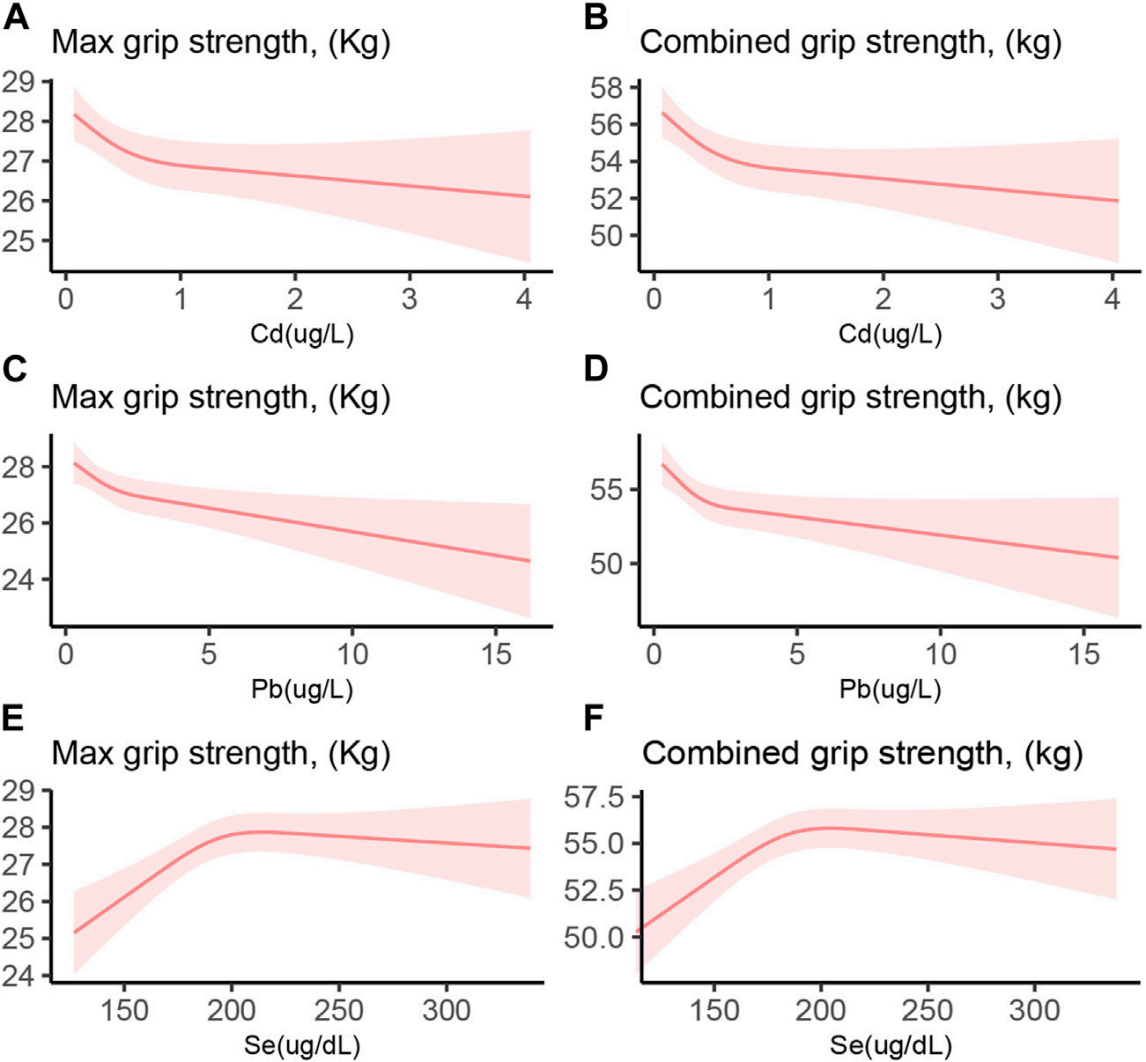
Restricted cubic spline of the association between Cd, Pb, and Se with HGS. **(A)** Cd and Max HGS **(B)** Cd and combined HGS **(C)** Pb and Max HGS **(D)** Pb and combined HGS **(E)** Se and Max HGS **(F)** Se and combined HGS. Red line and red transparent area represent smooth curve fit and 95% CI fit, respectively. Adjusted by age, gender, race, education levels, marital status, cotinine, drinking status, BMI, SBP, DBP, HbA1c, and TC.

Figure 2 depicts the relationship between the blood concentrations of Cd, Pb, and Se and HGS. A blood Se concentration exceeding approximately 200 μg/L does not further enhance HGS, demonstrating an inverted U-shaped relationship. Conversely, an increase in blood Pb concentration corresponded with a decline in HGS, indicating a direct inverse relationship. Supplementary Table S2 categorizes Se concentrations into four quartiles. Compared to the quartile with the lowest Se levels, the second quartile exhibited significantly enhanced HGS. However, the third and fourth quartiles did not exhibit comparable strength. This observation implies that excessively high Se concentrations may be detrimental to HGS.

### 3.3 Role of Se on the association between heavy metals (Cd and Pb) and HGS

**FIGURE 3:**
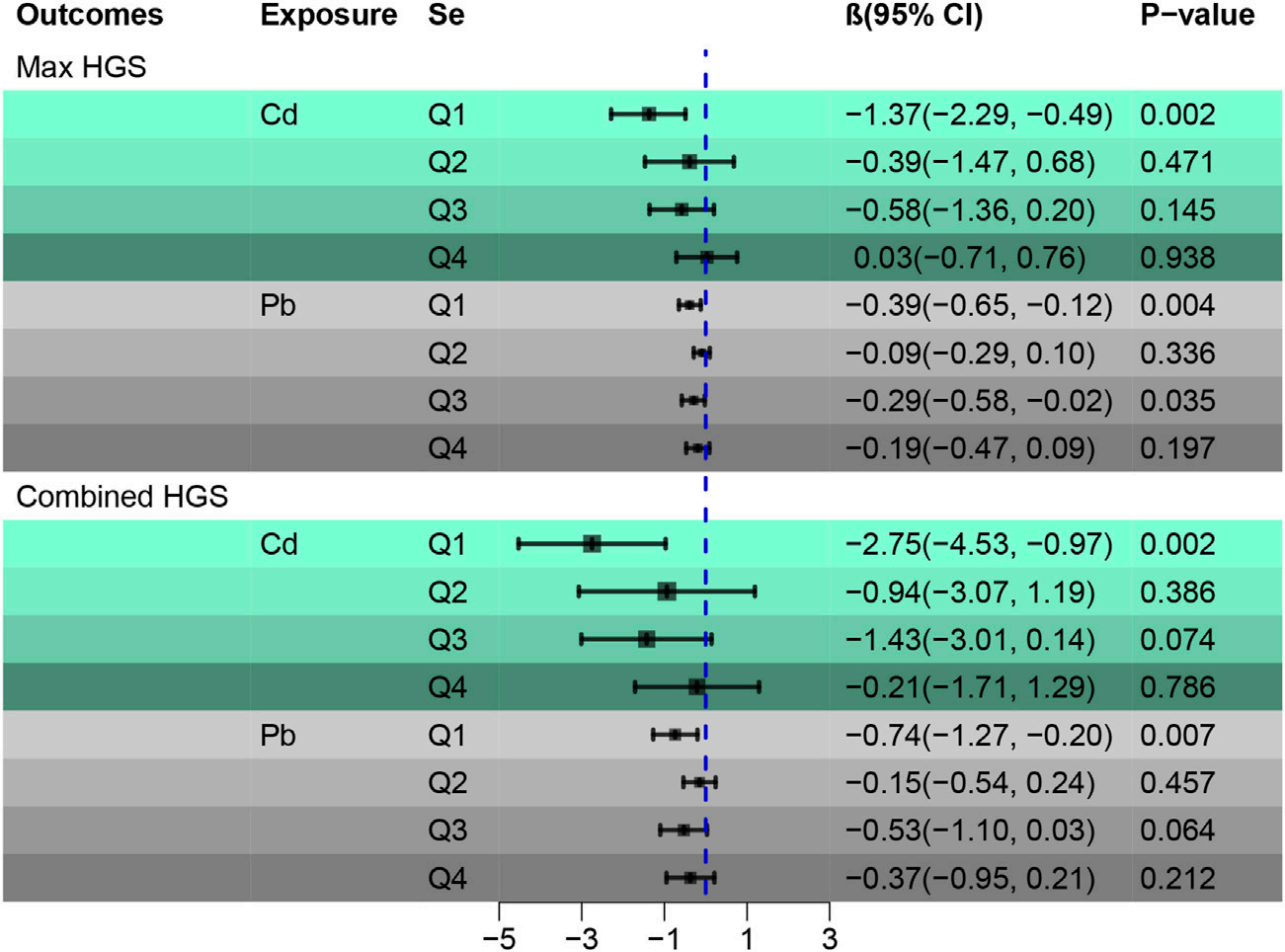
Stratified analysis of associations between Cd and Pb with HGS according to the quartiles of Se. Adjusted by age, gender, race, education levels, marital status, cotinine, drinking status, BMI, SBP, DBP, HbA1c, and TC.

Figure 3 provides a stratified analysis based on Se quartiles, examining the associations between Cd and Pb with HGS. It elucidates the interplay between blood concentrations of Cd and Pb with HGS across varying Se concentrations. For Max HGS, the deleterious impacts of Cd and Pb are attenuated with increasing Se concentrations. Significantly, participants within the second and fourth Se quartiles experienced the least harm from Cd on Max HGS. Similarly, the negative influence of Pb on Max HGS was substantially reduced within these Se quartiles. With regard to combined HGS, a consistent trend is observed: the adverse effects of both Cd and Pb recede as Se levels increase. Specifically, the second and fourth Se quartiles showcased participants least affected by Cd and Pb concerning combined HGS. For more detailed data, please refer to Supplementary Table S3.

Supplementary Table S4, using a general linear regression, delves deeper into the relationship between Se-Cd and Se-Pb exposures and grip strength. Taking the Se-Cd-group as a baseline, the β value for the Se + Cd + group falls between that of the Se + Cd- and Se- Cd + groups. This pattern implies that Se mitigates the detrimental impacts of Cd on Max HGS. An analogous pattern is discerned for Se-Pb, underscoring Se’s protective effect against the harmful influences of both Cd and Pb on HGS. These findings underscore the significant interplay between Se, Cd, and Pb, emphasizing Se’s potential protection role on HGS.

**FIGURE 4:**
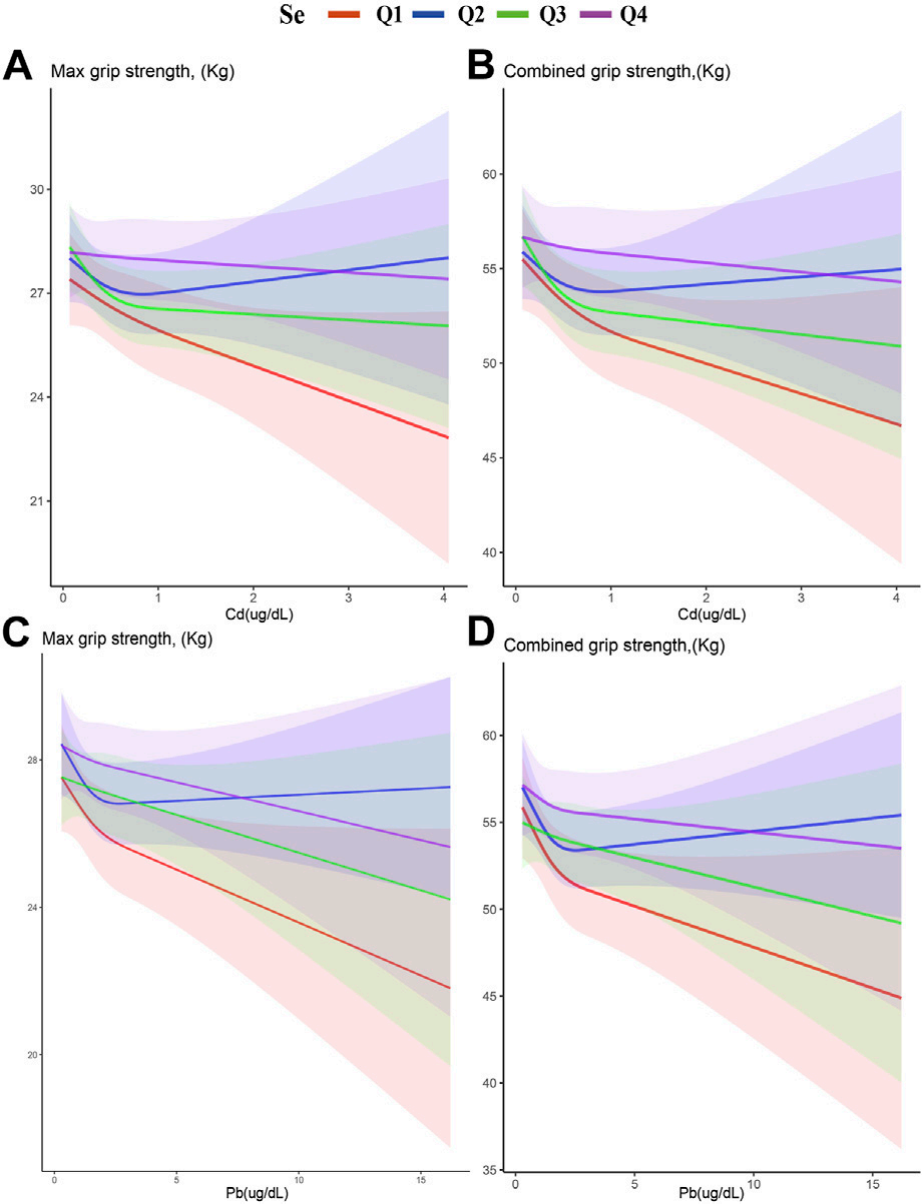
Stratified restricted cubic spline of the association of exposure and HGS according to the quartiles of Se. **(A)** Cd and Max HGS **(B)** Cd and combined HGS **(C)** Pb and Max HGS **(D)** Pb and combined HGS. Adjusted for age, gender, race, education levels, marital status, cotinine, drinking status, BMI, SBP, DBP, HbA1c, and TC. The red, blue, green, and purple lines represent the first, second, third, and fourth quartiles of Se, respectively. The shaded part represents the 95% confidence interval.

Figure 4 displays restricted cubic splines, stratified by Se quartiles. The negative correlation between Cd and Pb with HGS weakens by ascending Se levels. This attenuation is most notable between the first and second Se quartiles, where the second quartile shows the weakest correlation. Intriguingly, the fourth Se quartile reveals a resurgence of the inverse relationship between Cd and Pb with HGS. This pattern suggests that there exists an optimal range of Se concentrations wherein it most effectively mitigates the adverse impacts of Cd and Pb on HGS.

**FIGURE 5:**
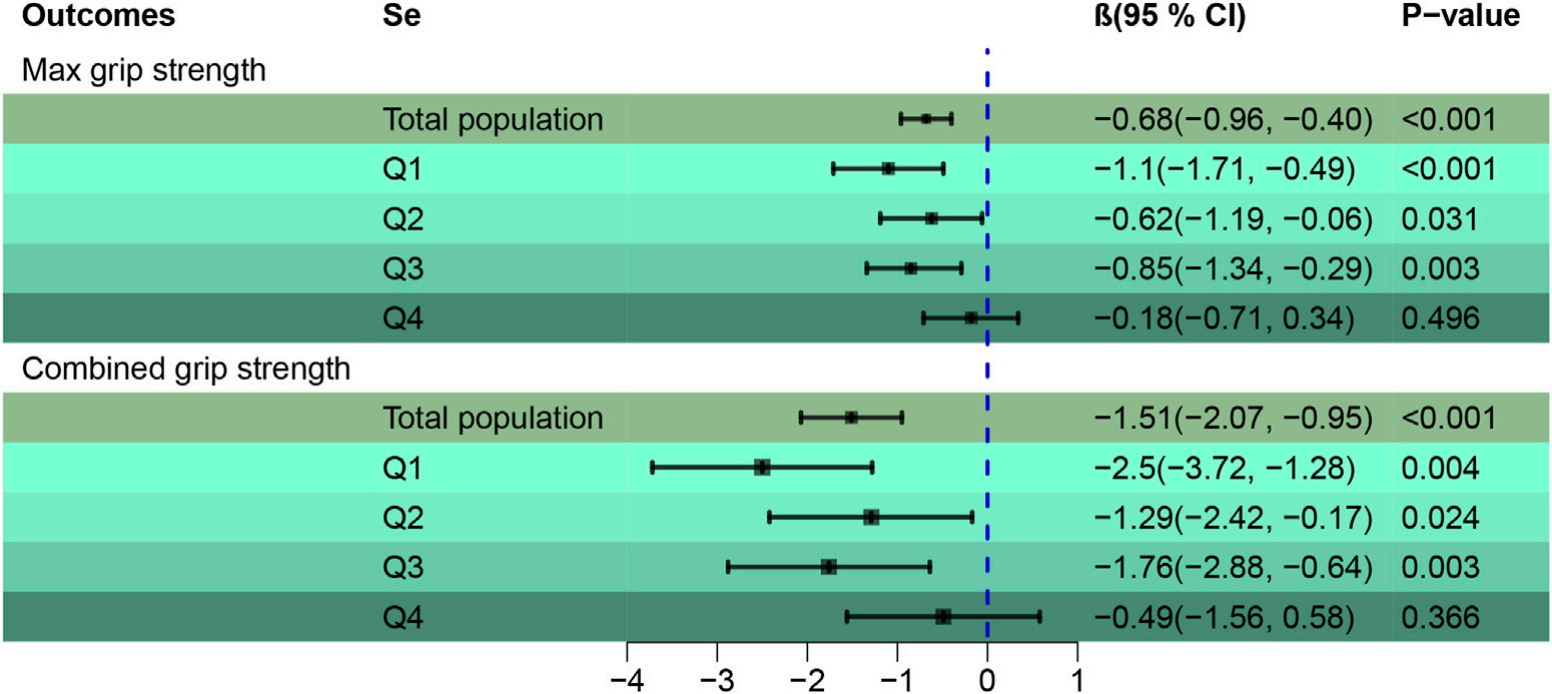
Qgcomp regression to assess the association of the mixture of blood metals (Cd, Pb) with HGS across Se quartiles. Adjusted by age, gender, race, education levels, marital status, cotinine, drinking status, BMI, SBP, DBP, HbA1c, and TC.

Figure 5 Employs the Qgcomp regression analysis to examine the relationship between mixed metal exposures (Cd, Pb) in the blood and HGS across various Se quartiles. A pronounced negative association was found between Cd and Pb mixtures with both Max (β = −0.68, 95% CI [-0.96, −0.40]) and combined HGS (β = −1.51, 95% CI [-2.07, −0.95]), establishing statistical significance (*p* < 0.001). Stratified Qgcomp regression reveals that the adverse effects of heavy metals on Max HGS are somewhat buffered when Se concentrations lie within the second and third quartiles, compared to the more pronounced effects observed in the first and fourth quartiles. This pattern indicates Se’s capacity to counteract the detrimental impacts of heavy metals on Max HGS. A similar trend was discerned for combined HGS. For more detailed data, please refer to Supplementary Table S5.

Moreover, Supplementary Table S6 delves into the correlations between a broader metal mixture in the blood (Se, Cd, Pb) and grip strength. Notably, apart from the observed association with Max HGS (*β* = −0.83, 95% CI [-1.53, −0.14]), other correlations did not reach statistical significance. Collectively, these insights emphasize Se’s potential protective role against the adverse influences of heavy metal exposures on HGS.

### 3.4 Subgroup analyses

In the subgroup analysis, additional adjustments were made for the “MET score and HEI-2015 index.” The detailed description of adjusted variables is provided in Supplementary Tables SA1, SA2.

The Qgcomp regression method examines the relationship between mixed blood metals (Cd and Pb) across different Se quartiles in relation to HGS. This method effectively stratifies, and groups data based on gender and age.

Supplementary Table S7 highlights gender-specific disparities. The composite of Cd and Pb shows a negative correlation with both Max and combined HGS. This negative association is more discernible among females. As Se levels rise, its protective effect against heavy metal-induced HGS impairment in females exhibits an initial strengthening followed by a subsequent weakening trend. In contrast, for males, a non-linear positive trend emerges, though it lacks statistical significance. These observations suggest that females might be more vulnerable to heavy metal-induced impairment in grip strength, and specific Se concentrations can counteract such effects.

Supplementary Table S8 dissects age-related variations. The combined effects of Cd and Pb are inversely correlated with both Max and combined HGS. However, this correlation is notably stronger among older individuals, suggesting a more pronounced detrimental impact of heavy metals on the elderly’s HGS. With the elevation of Se concentration, its capacity to mitigate the adverse effects of heavy metals on HGS in the elderly follows an initial strengthening followed by a subsequent weakening trend. Conversely, a non-linear positive trend emerges in the middle-aged group, although it does not achieve statistical significance. These findings emphasize the heightened susceptibility of the elderly to HGS reductions due to heavy metal toxicity and the protective role of Se within a specific concentration range.

Supplementary Table S9 detailed the analysis related to diabetes, categorizing participants into two groups based on glycated hemoglobin (HbA1c) levels: at risk of diabetes (HbA1c ≥ 6.5) and non-diabetic (HbA1c < 6.5). The study found that the combined effects of Cd and Pb on maximal and overall HGS were negatively correlated in both groups. However, a deeper stratified analysis using qgcomp regression revealed that in the diabetes risk group, only the lowest quartile (Q1) of Se (Se) showed a significant statistical correlation with HGS, while higher quartiles (Q2, Q3, Q4) did not show a significant association. In contrast, in the non-diabetic group, both Q1 and Q3 quartiles of Se were correlated with HGS, and a negative trend was observed across quartiles Q1 to Q4. These results underscore the limited protective role of Se in mitigating HGS decline caused by heavy metal toxicity in the diabetes risk group. Therefore, individuals at risk of diabetes should be particularly cautious about heavy metal-induced handgrip strength impairment. These findings provide important perspectives for future research, especially regarding the impact of heavy metal exposure on HGS in populations with different health statuses.

### 3.5 Sensitivity analyses

A sensitivity analysis was conducted wherein further adjustments were made for HGS-related variables. In the sensitivity analysis, additional adjustments were made for “MET score and HEI-2015 index.” This adjustment aimed to explore the relationship between both individual and combined blood metals (Cd and Pb) across different Se quartiles and HGS. This relationship was assessed using stratified analysis and the Qgcomp regression approach.

Supplementary Table S10 reveals that as Se concentrations escalate, its potential to offset the adverse impacts of individual heavy metals on HGS emerges as a non-linear positive trend, though this trend does not reach statistical significance. Conversely, Supplementary Table S11 indicates that the combination of Cd and Pb has an inverse relationship with both maximum and total grip strength. With increasing Se levels, its protective role against the negative effects of combined heavy metal exposure on HGS becomes evident, following a non-linear positive trajectory that is statistically significant.

## 4 Discussion

Environmental pollutants, especially heavy metals, severely impact human health. In this study, we observed a negative correlation between heavy metals in the blood, including Cd and Pb with HGS, while Se showed a positive correlation with HGS. However, it is noteworthy that this positive correlation diminishes when the concentration of Se in the blood exceeds about 200 ug/L. Only moderate levels of Se can mitigate the adverse effects of Cd and Pb on HGS. Therefore, focusing on the impact of heavy metals on HGS not only helps in identifying high-risk exposures to heavy metals and reducing environmental contact with them but also emphasizes the importance of appropriately supplementing with the Se element. This research provides crucial information for reducing HGS damage due to heavy metal exposure. To the best of our understanding, we demonstrated that both the higher and lower blood Se concentrations were detrimental to the middle and elderly’ HGS impairment related to Cd and Pb for the first time. Only moderate Se could mitigate the adverse effect of Cd and Pb on HGS.

### 4.1 Comparison with other studies

This study employed data from the NHANES spanning 2011 to 2014. It aimed to discern both individual and combined effects of Cd and Pb exposure levels in the blood on HGS among middle-aged and elderly. Additionally, the study probed the potential protective effect of Se supplementation on these relationships, shedding light on a new interplay between Se, Cd, and Pb, with HGS.

Historically, numerous studies have highlighted the health benefits of Se and the hazards of heavy metal toxicity. One study suggested that heightened Cd exposure corresponds with decreased HGS, underscoring the potentially harmful influence of heavy metals on HGS (García-Esquinas et al., 2020). Another study focusing on elderly women drew a correlation between low serum Se levels and diminished HGS, implying that Se supplementation might enhance HGS (Beck et al., 2007). However, investigations into Se’s countering effect on health issues induced by heavy metal toxicity remain limited. A recent cross-sectional study postulates that optimal Se levels can counter the toxic effects of Cd and Pb, particularly on lung function (Fan et al., 2023). This intimates that Se might play a pivotal role in mitigating health concerns stemming from heavy metal toxicity. The research findings align with our study’s principles and outcomes, underscoring the impact of heavy metal exposure on HGS. Adequate Se levels may mitigate the toxicity of these heavy metals by enhancing the activity of certain detoxifying enzymes. This Se -mediated effect should not be perceived as a direct barrier but rather as a biochemical mechanism that combats the toxicity of heavy metals at the cellular level.

### 4.2 Potential mechanism

Pb and Cd, as toxic metals with significant occupational health implications, are playing an increasingly important role in current environmental pollution studies. The impact of these metals on organs and biological systems is widespread and profound, capable of causing serious health problems, including acute and especially chronic poisoning symptoms. A potential correlation exists between blood levels of Se, Cd, and Pb with HGS, potentially arising from various underlying mechanisms.

#### 4.2.1 The toxic mechanisms of Cd and lead

Research findings reveal that Cd is an effective inducer of oxidative stress, DNA damage, endoplasmic reticulum (ER) stress, and autophagy. Excessive stress responses can lead to significant tissue damage by triggering various cellular death pathways such as apoptosis, ferroptosis, and necroptosis (Ma et al., 2022). Particularly, the kidneys, liver, bones, and nervous system, which are the main targets of chronic Cd exposure, are proven to be at increased risk of kidney damage (Barregard et al., 2022), liver injury (Bernhoft, 2013), neurodegenerative diseases (Ma et al., 2022), and osteoporosis (Luo et al., 2021) due to environmental and occupational Cd exposure. Similarly, environmental contamination with Pb is a global public health issue, associated with various diseases, especially neurodegenerative diseases, where microglia-mediated neuroinflammation plays a key role in lead’s neurotoxicity (Hu et al., 2023). Additionally, research indicates a negative correlation between Cd and Pb with HGS, suggesting that these heavy metals may impair HGS by affecting multiple physiological mechanisms.

The common mechanism of toxicity induced by heavy metals is believed to be due to their induction of excessive production of reactive oxygen species (ROS), depletion of antioxidants, and inhibition of antioxidant defense enzyme activities. This disrupts the cellular redox balance, leading to adverse health effects (Rehman et al., 2018; Balali-Mood et al., 2021). Epidemiological studies and laboratory data both indicate that heavy metal-induced oxidative stress is closely related to the cellular response to increased risks of tumors, neurological diseases, diabetes, infertility, developmental disorders, renal dysfunction, and cardiovascular diseases (Paithankar et al., 2021). In normal cellular metabolism, the production of reactive oxygen species (ROS) is regulated by the cellular antioxidant system to maintain a balanced state. The oxidative stress caused by Cd and Pb in the body, through inducing excessive production of ROS, leads to the imbalance of the cellular antioxidant regulatory system (Matović et al., 2015). Redox-inactive metals such as Cd, arsenic (As), and Pb exhibit their toxicity by binding to the sulfhydryl groups of proteins and depleting glutathione (Jomova and Valko, 2011; Jozefczak et al., 2012). Studies have found that increased blood Pb levels are associated with decreased serum levels of the anti-aging protein alpha-klotho (α-klotho), suggesting that Pb exposure may exacerbate oxidative stress by affecting the expression or activity of *a*-klotho, leading to aging manifestations like muscle loss, which may affect handgrip strength (Kim et al., 2022). Animal studies also show that Cd accumulation can lead to a decrease in the activity of antioxidant enzymes (such as T-SOD, GST, and GPX), causing myocardial inflammation (Guo et al., 2020).

Cellular toxicity caused by heavy metal exposure has been confirmed in numerous studies (Paithankar et al., 2021) For instance, Cd can induce apoptosis in bronchial epithelial cells by activating the mitochondria-mediated intrinsic apoptotic pathway (Cao et al., 2021). Under higher concentrations of Cd exposure, kidney cells suffer damage, characterized by mitochondrial injury, cell swelling, and loss of basement membrane structure, while long-term low concentration exposure can lead to renal fibrosis (Thijssen et al., 2007). In the hypoxic environment associated with chronic obstructive pulmonary disease (COPD), Cd exacerbates damage to the kidneys and bones by overexpressing DMT-1, increasing the risk of COPD-related complications (Cirovic et al., 2022), while higher COPD incidence and mortality rates are associated with reduced HGS (Holden et al., 2021). Cd and Pb also disrupt the bioenergetics of human osteoblasts, reducing ATP production, mitochondrial complex activity, and aerobic respiration, and diminishing antioxidant defense enzyme activity and intracellular glutathione levels (Al-Ghafari et al., 2019). Exposure to Cd also induces oxidative stress, depletes cellular thiols, and stimulates apoptosis in mouse myoblasts (Zhang et al., 2019). Cd and Pb interfere with the normal function of calcium ions in cells, particularly affecting calcium-dependent proteins like TnC, disrupting intracellular metabolism and muscle fiber function, which may lead to metabolic disorders and toxic effects (Chao et al., 1990). Smoking-induced exposure to Cd and Pb can cause endothelial cells to release inflammatory and atherosclerosis-related cytokines, damaging endothelial cell processes and morphology, and weakening their protective capabilities (van Strijp et al., 2023). Animal experiments show that the toxic effects of Pb on muscles include disrupting normal glucose metabolism, altering key enzyme activities and energy metabolism, potentially causing damage to muscle tissue structure, impacting muscle function and quality, and negatively affecting overall health (Das et al., 2020). Another animal study suggests that Pb exposure’s damage to skeletal muscle function mainly includes reduced muscle mass, decreased exercise capacity, weakened muscle strength, disrupted lipid metabolism, increased inflammatory responses, and upregulated expression of pro-apoptotic genes, possibly due to lead’s direct effects on muscle cell structure, metabolism, and inflammatory pathways (He et al., 2023).

A study focusing on residents near mining and smelting areas in Northwest China showed that the combined exposure to Cd and Pb is associated with systemic immune-inflammatory responses, which intensify with increasing levels of Cd and Pb co-exposure (Zhang et al., 2022). In a study on children, it was found that exposure to heavy metals such as Pb and Cd could disrupt the body’s immune balance and induce inflammatory responses, adversely affecting health (Zhang et al., 2020). The toxic effects of Cd operate by disrupting normal cellular signaling pathways, particularly by activating the C/EBP-DDIT3 signaling pathway, inducing endoplasmic reticulum (ER) stress and inflammatory responses, damaging the normal functions of airway cells, and potentially leading to various pulmonary diseases such as chronic obstructive pulmonary disease (COPD) (Kim et al., 2017). Cd in tobacco smoke can activate inflammatory responses through free radical-mediated tissue damage (Milnerowicz et al., 2015). Another animal study indicated that the damage Cd causes to skeletal muscles mainly manifests as decreased muscle weight and strength, reduced exercise capacity, disrupted lipid metabolism, enhanced inflammatory responses, and increased expression of pro-apoptotic genes. These damages likely stem from Cd’s direct toxic effect on muscle cells and the resulting inflammatory responses (He et al., 2023).

Pb is widely recognized as a potent neurotoxin, and a substantial body of research evidence suggests that exposure of the developing central nervous system (CNS) to Pb can lead to changes in cognition, motor skills, and behavior. Although its neurotoxic mechanisms are complex, Pb appears to alter the release of neurotransmitters, cause excitotoxicity, and ultimately lead to apoptosis (Stretesky and Lynch, 2004; Toscano and Guilarte, 2005). Epidemiological and clinical research data indicate that Cd can penetrate the blood-brain barrier, infiltrate the nervous system, and cause a range of severe neurological symptoms such as headaches, dizziness, olfactory disturbances, memory loss, polyneuropathy, attention deficits, and Parkinson-like symptoms (Viaene et al., 2000; Ciesielski et al., 2013). Exposure to Pb and Cd interferes with key processes in adult neurogenesis, inducing neurotoxicity by affecting adult neurogenesis and accelerating or exacerbating cognitive decline associated with neurodegenerative and/or aging processes (Wang and Matsushita, 2021). The intake of Cd and Pb affects the homeostasis and neuroprotective cascade reactions of astrocytes, including glutamate/GABA-glutamine transport, antioxidant mechanisms, and energy metabolism. Abnormalities in astrocyte pathways may promote or trigger neurodegenerative changes (Li et al., 2021). In an animal study, it was found that a mixture of Cd and Pb induced synergistic toxicity in astrocytes, potentially damaging the blood-brain barrier (BBB) and leading to behavioral disorders in developing rats, such as hyperactivity, enhanced grip strength, and learning and memory deficits (Rai et al., 2010).

The common characteristic in the mechanisms by which Cd and Pb damage grip strength is their ability to induce oxidative stress in the body. This is accomplished by the production of excessive reactive oxygen species (ROS), leading to an imbalance in the cellular antioxidant regulatory system. This redox imbalance not only damages cellular structures and functions, including muscle cells, but can also directly affect grip strength. The cytotoxicity caused by Cd and lead, through various mechanisms such as mitochondria-mediated apoptosis and cell membrane damage, affects cell survival, which is crucial for maintaining the health of muscle cells as their functional state is directly linked to muscle strength. Studies suggest that Cd and lead, by affecting the structure and metabolic pathways of muscle cells, may reduce muscle function and quality, thereby weakening grip strength. Additionally, these metals can induce immune and inflammatory responses, which also adversely affect the health and functionality of muscle tissue. These findings highlight the importance of further research into the exposure to Cd and Pb and their impact on muscle health.

In terms of the differences in the mechanisms by which Cd and Pb damage grip strength, Pb is widely regarded as a potent neurotoxin, with particularly significant effects on the central nervous system. Pb exposure may alter the release of neurotransmitters and nerve conduction, which could indirectly affect the neural control of muscles, thereby impacting grip strength. In comparison, while Cd does affect the nervous system, its impact might not be as direct or pronounced as that of lead. Cd primarily affects the kidneys, liver, bones, and nervous system, and its damage may indirectly affect overall health status and muscle function through the impairment of these organs’ functions. Pb is known for its significant damage to the nervous system, potentially causing direct effects on muscles by disrupting normal glucose metabolism processes and energy metabolism mechanisms. In contrast, the effects of Cd might be more related to the oxidative stress and cytotoxicity it induces. These differences reflect the complexity of the mechanisms of action of these metals within the body and suggest that future research needs to delve deeper into how these metals affect muscle function and overall health through different pathways.

The impact of Cd and Pb on HGS may involve a range of complex mechanisms, including oxidative stress, cytotoxicity, immune responses, and inflammation. Although both metals exhibit similarities in certain toxic mechanisms, they also show significant differences, particularly in their effects on the nervous system and specific metabolic pathways. Understanding these similarities and differences is crucial for the prevention and treatment of health issues caused by these heavy metals. Given these findings, the current focus should be on addressing the adverse effects of Cd and Pb on HGS and implementing appropriate preventive and intervention measures. This requires not only application in clinical medicine but also attention in public health policies and environmental management to reduce the exposure risk to these heavy metals and protect human health.

#### 4.2.2 The protective mechanism of Se

Se, as a crucial trace element, plays an essential role in human health, especially in antioxidant activities and maintaining immune functions. When exploring how Se can counteract the damage caused by Cd and Pb to HGS, several potential protective mechanisms can be considered.

Se plays a crucial role in enhancing the cell’s antioxidant defense system and maintaining redox balance, which helps alleviate the damage caused by oxidative stress from Cd and Pb on muscle cells, thereby protecting HGS. Se, by improving antioxidant defense, immune function, and metabolic homeostasis, can reshape progressive and spontaneous physiological changes caused by oxidative stress, potentially preventing diseases and promoting healthy aging (Bjørklund et al., 2022). As a component of selenoproteins, the trace element Se performs an important antioxidant function in preventing muscle tissue damage (Pei et al., 2023). Additionally, Se enhances the activity of antioxidant enzymes, with its physiological effects primarily stemming from its role in selenoproteins (Avery and Hoffmann, 2018). Se -containing glutathione peroxidases help reduce damage caused by free radical reactions (Kieliszek and Błażejak, 2016). Se -dependent glutathione peroxidases (GPX1-4 and GPX6) and thioredoxin reductases (TrxR1-3) can directly inhibit oxidative stress. Cytoplasmic GPX4 is crucial for embryonic development and cell survival, while GPX1 is the primary metabolic form in the human body to combat severe oxidative stress (Wu et al., 2012). GPX1 maintains redox balance by detoxifying reactive oxygen species (ROS) (Avery and Hoffmann, 2018). Studies suggest that ROS accumulated over a lifetime may lead to muscle loss, and selenoproteins like glutathione peroxidases can protect muscle cells from oxidative damage (Fernández-Lázaro et al., 2020).

Se has potential protective effects in preventing cell damage caused by heavy metals. *In vitro* studies show that the presence of Se in tissues can reduce the toxicity of Cd not only by storing and redistributing these toxic metals but also by forming Cd-Se complexes and binding with selenoprotein P, thereby decreasing the bioavailability of Cd (Siscar et al., 2014). Furthermore, Se may promote cellular repair and regeneration. By regulating intracellular metabolic processes, Se helps alleviate the toxic effects of heavy metals. It may assist in restoring metabolic imbalances caused by Cd and lead, maintaining the normal function of muscle cells, and thus preserving handgrip strength. For example, Se can regulate signaling pathways affecting cell survival to protect cardiac muscle cells (Shalihat et al., 2021). Se plays a crucial role in the regulation of thyroid hormones, controlling normal development, growth, and cell metabolism. These studies indicate that Se not only functions in antioxidant defense but also plays an important role in maintaining cellular structure and function, as well as promoting damage repair (Rosen and Liu, 2009).

Se may protect handgrip strength by alleviating inflammatory responses caused by Cd and Pb and promoting the repair processes post-inflammation. In the human body, Se primarily exists in the form of selenocysteine and selenoproteins, among which selenoproteins, acting as antioxidants, have significant anti-inflammatory effects. Particularly, one of the major selenoproteins, glutathione peroxidase (GPX), can effectively control the excessive production of free radicals at inflammation sites. Additionally, selenoprotein-S also plays an important role in regulating inflammatory cytokines (Hariharan and Dharmaraj, 2020). Through these mechanisms, Se helps to reduce the inflammatory response caused by heavy metals, alleviating damage to muscle tissue, thus aiding in maintaining or restoring normal muscle function and handgrip strength.

While research on Se‘s protective mechanisms against the neurotoxicity caused by heavy metals like Pb and Cd is not yet extensive, existing studies have shown that Se can significantly delay or mitigate certain neurotoxic effects of methylmercury, thus protecting motor and sensory functions (Heath et al., 2010). An animal study indicated that Se could alleviate the decline in glutathione levels and the reduction in acetylcholinesterase activity caused by arsenic. It also reduced oxidative stress and the production of reactive oxygen and nitrogen species, as well as lowered inflammation parameters. Overall, Se effectively mitigated behavioral disorders in rats caused by arsenic through its anti-inflammatory, antioxidant, and anti-apoptotic mechanisms (Adedara et al., 2020). The synergistic action of these mechanisms is crucial for protecting the nervous system from the adverse effects of heavy metals. Thus, the potential role of Se in neuroprotection deserves further investigation, especially its potential in mitigating neural damage caused by exposure to heavy metals.

In summary, Se exerts a protective effect by reducing the adverse impact of Cd and Pb on handgrip strength through its antioxidant properties, regulation of cellular metabolism, alleviation of inflammatory responses, and mitigation of neurotoxicity. This multifaceted protective role makes Se an important trace element in combating muscle damage caused by heavy metals. Consequently, judicious Se supplementation could enhance HGS. Nevertheless, an overabundance of Se may induce toxicity, potentially due to excessive Se triggering oxidative stress and generating harmful radicals that inflict cellular and organ damage. Se may also convert into deleterious selenite salts, perturbing cellular processes and producing toxic consequences. It is paramount to recognize that Se’s antagonistic efficacy is contingent upon intake levels and individual variabilities. As such, when leveraging Se’s antagonistic potential, its dosage and ensuing effects warrant meticulous consideration, and adjustments should be made attuned to specific circumstances. In essence, while Se offers a promising avenue to counteract the toxic effects of Cd and Pb, it should not be solely relied upon as a panacea. The impact of Cd and Pb on HGS is a complex process, involving multiple biological pathways. Se, as an important trace element, shows significant potential in countering the toxicity of these heavy metals. The findings of this research emphasize the importance of considering trace element interactions in environmental health studies and provide new perspectives for future research in this field. Future studies should further explore the interactions between Se and other trace elements, as well as how they collectively influence the impact of heavy metal exposure on human health.

### 4.3 Explanation for the subgroup analyses results

The subgroup analysis underscored the pronounced impact of gender and age on the association between HGS and blood metal mixtures (comprising Cd and Pb) across different Se quartiles. With respect to gender, females exhibited greater vulnerability to the negative implications of heavy metals on HGS. An increase in Se concentration notably mitigated this damage, and the protective effect began to diminish after reaching a certain threshold, demonstrating an initial enhancement followed by a decline. Conversely, males exhibited a nonlinear negative correlation, although it lacked statistical significance. Such observations accentuate the differential gender-based effects of heavy metal exposure on HGS.

In terms of age, the elderly were notably more vulnerable to the adverse effects of heavy metals on HGS. With the increase in Se concentration, there is an initial strengthening followed by a subsequent weakening trend in Se’s ability to mitigate the adverse effects of heavy metals on HGS in the elderly. For the middle-aged group, a discernible nonlinear negative trend was observed, but, akin to males, it did not attain statistical significance. This emphasizes the paramount role age assumes in the interplay between heavy metal exposure and HGS.

For populations at risk of diabetes, these findings highlight that the protective role of Se in mitigating the decline in HGS caused by heavy metal toxicity may be limited. This phenomenon suggests that in individuals with a risk of diabetes, the protective effect of Se against heavy metal-induced handgrip strength impairment might not be as significant as in those without a diabetes risk. Therefore, for people at risk of diabetes, it becomes even more crucial to avoid the impairment of grip strength caused by heavy metals. Furthermore, this indicates that future research should delve deeper into how the risk of diabetes influences the toxicity of heavy metals and the protective role of Se, as well as how to protect this specific population more effectively from the adverse effects of heavy metals.

The subgroup analysis of this study reveals the importance of considering factors such as gender, age, and diabetes risk when assessing the relationship between heavy metal (Cd and lead) exposure and HGS. This underscores the need to consider the variability in susceptibility and response to heavy metal exposure among different demographics and risk groups, offering a more nuanced understanding of the potential health impacts. These insights are invaluable for shaping future interventional approaches. The results illuminate the nuanced susceptibilities in HGS impairments, underscoring that, within specific concentration limits, Se effectively counteracts the negative effects of heavy metals on HGS. These insights serve as a foundational academic base to inform and refine targeted preventive and healthcare strategies.

### 4.4 Clinical implications

This study elucidates the pivotal relationship between Se and the adverse effects of Cd and Pb on HGS, offering substantial implications for clinical contexts. It underscores the imperative of risk assessment and management, particularly when individuals present with compromised grip strength in areas with heightened heavy metal levels. Such findings accentuate the importance of targeted interventions and the broader dissemination of public health advisories. Considering the heightened susceptibility of females, elderly individuals, and diabetes patients to the toxicity of heavy metals, special attention should be given to these populations. When formulating proactive measures, risk variations associated with gender, age, and diabetes patients should be considered. Moreover, policy recommendations could push for stringent controls on heavy metal exposure, especially in settings predominantly occupied by these vulnerable groups. A deeper dive into research is requisite to delineate the causal dynamics among Se, Cd, and Pb with HGS, and to unpack their intertwined biological mechanisms.

In essence, Se stands as a potent counteragent to the toxicity induced by heavy metals such as Cd and Pb. This provides a robust academic foundation to mitigate HGS afflictions and amplify preventive awareness. It is pivotal to understand that while Se can play a therapeutic role in neutralizing heavy metal toxicity, its intake should be judicious. A balanced Se intake can be adjunctive in treatment, emphasizing the salience of routinely monitoring heavy metal levels in the body. This facilitates early identification of potential toxic threats. However, any intervention should be judiciously overseen by healthcare professionals to ensure Se consumption remains within safe limits.

### 4.5 Limitations and strengths

The study’s foremost strength stems from its application of multiple models to probe the effects of both isolated and combined exposures to Se, Cd, and Pb with HGS, thereby amplifying the research’s breadth and depth. Bolstering the study’s credibility, it leveraged the nationally representative NHANES dataset, benefitted from a substantial sample size, and meticulously adjusted for numerous potential confounders, which consolidates the trustworthiness of its conclusions.

However, the research is not devoid of limitations. The study’s cross-sectional design inhibits discerning a definitive causal link between Se, Cd, and Pb with HGS. Even with rigorous accounting for known confounders, the potential influence of unquantified or overlooked confounding factors cannot be dismissed. The assessment of HGS, while insightful, has inherent limitations such as localization, singular indices, individual variations, testing inaccuracies, physiological shifts, and extrinsic influences, potentially introducing biases. Another point of contention is the generalizability of the findings. Since the data primarily pertains to American adults, extrapolating these conclusions to global populations is circumspect. Although several studies have ventured into the relationships between Se, Cd, or Pb and functions like lung capacity, comprehensive human studies that integrate all these elements remain elusive. The intricate interplay and mechanisms of Se’s interaction with bloodborne heavy metals, such as Cd and Pb, are yet to be demystified. As a forward-looking recommendation, the validation of this study’s conclusions would benefit immensely from longitudinal cohort studies boasting expansive and diverse sample sizes. The absence of forearm circumference data is a limitation in our study, as it could have a direct biological correlation with HGS. Therefore, not including this variable might limit our comprehensive understanding of factors influencing HGS. Secondly, iron, a key element for hemoglobin synthesis, is crucial for maintaining normal muscle function and strength. Both iron deficiency and excess can adversely affect muscle health, thereby indirectly impacting handgrip strength. However, our study did not include data on iron levels, suggesting that future research should consider iron levels to understand the impact of different nutrients and environmental factors more fully on handgrip strength. Lastly, the combined effects of Se with other trace elements, such as zinc and copper, which may have similar or superior biological effects, might be more effective in improving HGS. This indicates that future research should more comprehensively consider and investigate the interactions and synergistic effects of various trace elements to gain a deeper understanding of how these elements collectively influence muscle function and handgrip strength.

## 5 Conclusion

This study primarily investigated the effects of Se, Cd, and Pb on HGS. The results showed that, after adjusting for confounding factors, Se was positively correlated with HGS, while Cd and Pb were negatively correlated. Further analysis revealed that higher levels of Se could mitigate the negative impact of Cd and Pb on HGS. Additionally, there was an inverted U-shaped relationship between Se and HGS, indicating that the benefits of Se on HGS tend to plateau beyond approximately 200 μg/L. Stratified analysis revealed that as Se levels increased, the negative effects of Cd and Pb on HGS gradually diminished. However, in populations at high risk of diabetes, the role of Se in counteracting the HGS decline induced by heavy metals was limited, suggesting that more detailed management of heavy metal exposure is needed in this group. The study underscores the important role of Se in alleviating the adverse effects of Cd and Pb on HGS, particularly its potential protective effects in specific high-risk groups. This provides a new perspective for future research to explore optimal strategies for Se supplementation and its synergistic effects with other nutritional and environmental factors.

## Data Availability

Publicly available datasets were analyzed in this study. This data can be found here: https://www.cdc.gov/nchs/nhanes/index.htm.
