# Supplementary material for: Moderate selenium mitigates hand grip strength impairment associated with elevated blood cadmium and lead levels in middle-aged and elderly individuals: insights from NHANES 2011–2014
Source: Front Pharmacol. 2023 Dec 14;14:1324583. doi: 10.3389/fphar.2023.1324583 (PMC10757617; doi:10.3389/fphar.2023.1324583)
Supplement: Supplementary file 1 [file Table1.DOCX]

Supplementary Material

Moderate selenium alleviates the hand grip strength impairment induced by cadmium and lead in middle-aged and elderly individuals: evidence from HNANES 2011-2014

Yafeng LIANG^1†^, Junqi WANG^2†^, Tianyi WANG^3^, Hangyu LI^4^, Chaohui YIN^5^, Jialin LIU^6^, Yulong WEI^7^, Junxing FAN^8^, Shixing FENG^469*^ and Shuangqing ZHAI^10*^

1 Beijing University of Chinese Medicine, Beijing, China.

2 Dongzhimen Hospital, Beijing University of Chinese Medicine, Beijing, China.

3 School of Management, Beijing University of Chinese Medicine, Beijing, China.

4 School of Life and Science, Beijing University of Chinese Medicine, Beijing, China.

5 School of Resources and Environment, Henan Agricultural University, Zhengzhou, Henan, China.

6 Dongfang Hospital, Beijing University of Chinese Medicine, Beijing, China.

7 School of Acupuncture-Moxibustion and Tuina, Beijing University of Chinese Medicine, Beijing, China.

8 Henan Provincial Health Talent Center, Zhengzhou, Henan, China.

9 Centre France Chine de la Médecine Chinoise, Selles sur Cher, France.

10 School of Traditional Chinese Medicine, Beijing University of Chinese Medicine, Beijing, China.

†These authors contributed equally to this work and share first authorship.

*** Correspondence:**

Shixing FENG

[fsx@bucm.edu.cn](mailto:fsx@bucm.edu.cn)

Shuangqing ZHAI
[zsq2098@163.com](mailto:zsq2098@163.com)

# Supplementary Data

### 1.1 Supplementary descriptions for the metabolic equivalent of task (MET) score[1]

#### **Table A1 Definition of the MET Score**

| Type of sports | Score |
| --- | --- |
| Vigorous work-related activity | 8.0 |
| Moderate work-related activity | 4.0 |
| Walking or bicycling for transportation | 4.0 |
| Vigorous leisure-time physical activity | 8.0 |
| Moderate leisure-time physical activity | 4.0 |

The MET score is calculated as shown below:

$$MET score = Suggested MET scores * {Number of days}^{a} *\mathrm{Minutes}^{b}$$

a: In a typical week

b: On a typical day

### 1.2 Supplementary descriptions for Healthy Eating Index (HEI)-2015

The Healthy Eating Index (HEI) is a scoring metric that can be used to determine overall diet quality as well as the quality of several dietary components. The HEI is a measure of diet quality, independent of quantity, that can be used to assess alignment with the Dietary Guidelines for Americans (DGA). Since 2005, researchers from the U.S. Department of Health and Human Services’ National Cancer Institute (NCI) and the U.S. Department of Agriculture (USDA) Center for Nutrition Policy and Promotion have collaborated to update the HEI based on updates to the DGA[2].

The HEI-2015 was designed to align with the 2015-2020 DGA. As with the previous DGA, the 2015-2020 edition emphasizes a variety of food groups, nutrient density, and improving food and beverage choices within calorie needs. After revisions, the HEI-2015 contains 13 components that sum to a total maximum score of 100 points. The total score is the sum of the score of adequacy components (i.e., foods to eat more of for good health) and moderation components (i.e., foods to limit for good health). The scoring criteria for each component are shown below[3].

#### **Table A2 Detailed description of HEI–2015^[1](https://epi.grants.cancer.gov/hei/developing.html#f1b)^ Components & Scoring Standards.**

| Component | Maximum points | Standard for maximum score | Standard for minimum score of zero |
| --- | --- | --- | --- |
| Adequacy: | | | |
| Total Fruits^[2](https://epi.grants.cancer.gov/hei/developing.html#f2b)^ | 5 | ≥0.8 cup equiv. per 1,000 kcal | No Fruits |
| Whole Fruits^[3](https://epi.grants.cancer.gov/hei/developing.html#f3b)^ | 5 | ≥0.4 cup equiv. per 1,000 kcal | No Whole Fruits |
| Total Vegetables^[4](https://epi.grants.cancer.gov/hei/developing.html#f4b)^ | 5 | ≥1.1 cup equiv. per 1,000 kcal | No Vegetables |
| Greens and Beans^[4](https://epi.grants.cancer.gov/hei/developing.html#f4b)^ | 5 | ≥0.2 cup equiv. per 1,000 kcal | No Dark Green Vegetables or Legumes |
| Whole Grains | 10 | ≥1.5 oz equiv. per 1,000 kcal | No Whole Grains |
| Dairy^[5](https://epi.grants.cancer.gov/hei/developing.html#f5b)^ | 10 | ≥1.3 cup equiv. per 1,000 kcal | No Dairy |
| Total Protein Foods^[6](https://epi.grants.cancer.gov/hei/developing.html#f6b)^ | 5 | ≥2.5 oz equiv. per 1,000 kcal | No Protein Foods |
| Seafood and Plant Proteins^[6](https://epi.grants.cancer.gov/hei/developing.html#f6b),[7](https://epi.grants.cancer.gov/hei/developing.html#f7b)^ | 5 | ≥0.8 oz equiv. per 1,000 kcal | No Seafood or Plant Proteins |
| Fatty Acids^[7](https://epi.grants.cancer.gov/hei/developing.html#f7b)^ | 10 | (PUFAs + MUFAs)/*SFAs* ≥2.5 | (PUFAs + MUFAs)/SFAs ≤1.2 |
| Moderation: | | | |
| Refined Grains | 10 | ≤1.8 oz equiv. per 1,000 kcal | ≥4.3 oz equiv. per 1,000 kcal |
| Sodium | 10 | ≤1.1 gram per 1,000 kcal | ≥2.0 grams per 1,000 kcal |
| Added Sugars | 10 | ≤6.5% of energy | ≥26% of energy |
| Saturated Fats | 10 | ≤8% of energy | ≥16% of energy |

1: The HEI-2020 components and scoring standards are the same as the HEI-2015. Intakes between the minimum and maximum standards are scored proportionately. The total HEI score is the sum of the adequacy components (i.e. foods to eat more of for good health) and moderation components (i.e. foods to limit for good health).

2: Includes 100% fruit juice.

3: Includes all forms except juice.

4: Includes legumes (beans and peas).

5: Includes all milk products, such as fluid milk, yogurt, and cheese, and fortified soy beverages.

6: Includes seafood, nuts, seeds, soy products (other than beverages), and beans, peas, and lentils.

7: Ratio of poly- and monounsaturated fatty acids (PUFAs and MUFAs) to saturated fatty acids (SFAs). PUFAs: polyunsaturated fatty acid; MUFAs: monounsaturated fatty acid; SFAs: saturated fatty acid.

**1.3 Supplementary methods for the selected covariates**

#### **Table A3 Detailed description of selected covariates**

| Covariates | Detailed description |
| --- | --- |
| Demography |  |
| Age | Recorded the middle-aged and elderly’s age at the time of the interview as a continuous variable with a minimum unit of 1 year. |
| Gender | Recorded the sex of the middle-aged and elderly at the time of the interview. It is divided into male and female. Male=1; Female=0. |
| Race | This is the race-ethnicity variable. Mexican American=1; Other Hispanic=2; Non-Hispanic White=3; Non-Hispanic African American=4; Other Race - Including Multi-Racial=5. |
| Marital status | The marital status question was asked of persons 14 years of age and older. Due to disclosure risks, marital status is only released for persons 20 years of age and older. Unmarried includes widowed, divorced, separated, never married, living with partner. Unmarried =0; married=1. |
| Education | This variable is the highest grade or level of education completed by adults 20 years and older. The response categories are less than 9th grade education, 9-11th grade education (includes 12th grade and no diploma), High school graduate/GED, some college, or associates (AA) degree, and college graduate or higher. Bachelor's degree below=0; bachelor’s degree and above=1. |
| Behaviours factors |  |
| Drinking | The alcohol drinking status of respondents was recorded and divided into drinkers (yes) and those who don’t drank (no). |
| MET score | See supplementary material 1.1 for details |
| HEI-2015 | See supplementary material 1.2 for details |
| Biomarkers |  |
| BMI | BMI was calculated as weight in kilograms divided by height in meters squared, and then rounded to one decimal place. |
| SBP | Systolic blood pressure, the top number, measures the force the heart exerts on the walls of the arteries each time it beats. |
| DBP | Diastolic blood pressure, the bottom number, measures the force the heart exerts on the walls of the arteries in between beats. |
| Cotinine | Cotinine is one of the primary metabolites of nicotine. The concentrations of cotinine in body fluids can be used as markers for active smoking and as indices for secondhand smoke (SHS) exposure. |
| HbA1c | Haemoglobin A1c (glycohemoglobin), a diabetes test that reflects plasma glucose for the previous 120 days, has been used to monitor diabetes for many years. |
| TC | Total cholesterol is the total amount of cholesterol in your blood. |

Abbreviations: BMI, body mass index; SBP, systolic blood pressure; DBP, diastolic blood pressure; TC, total cholesterol; MET, metabolic equivalent of task; HEI, healthy eating index.

**1.4** **Supplementary Explanation on the Detailed Measurement Process of Selenium, Cadmium, and Lead.**

Whole blood lead (Pb), cadmium (Cd) , total mercury (THg) manganese (Mn), and selenium (Se) concentrations are determined using inductively coupled plasma mass spectrometry. This multi-element analytical technique is based on quadrupole ICP-MS technology. Coupling radio frequency power into a flowing argon stream seeded with electrons creates the plasma. Predominate species in the plasma are positive argon ions and electrons. Diluted whole blood samples are converted into an aerosol using a nebulizer inserted within a spray chamber. A portion of the aerosol is transported through the spray chamber and then through the central channel of the plasma, where it experiences temperatures of 6000–8000 oK. This thermal energy atomizes and ionizes the sample. The ions, along with the argon, enter the mass spectrometer through an interface that separates the ICP, operating at atmospheric pressure (approximately 760 torr), from the mass spectrometer, operating at approximately 10-5 torr. The mass spectrometer permits detection of ions at each mass-to-charge ratio in rapid sequence, allowing individual isotopes of an element to be determined. Once inside of the mass spectrometer, the ions pass through the ion optics, then the mass analyzing quadrupole before being detected as they strike the surface of the detector. The ion optics focuses the ion beam using an electrical field.

 Electrical signals resulting from the detection of the ions are processed into digital information that is used to indicate the intensity of the ions and subsequently the concentration of the element. In this method blood samples are diluted with 18 M-ohm water and with diluent, containing 1% v/v tetramethylammonium hydroxide (TMAH), 0.05% disodium ethylenediamine tetraacetate (EDTA), 5% ethyl alcohol, 0.05% Triton X-100?, Au is added to reduce intrinsic Hg memory effects, Rh for internal standardization of Cd, and Bi for internal standardization of Hg and Pb. The samples were prepared with the following ratio Sample: Water: Diluent = 1:1:48 correspondingly.

## References

1. Edwards TC, Guest B, Garner A, Logishetty K, Liddle AD, Cobb JP. The metabolic equivalent of task score : a useful metric for comparing high-functioning hip arthroplasty patients. *Bone Joint Res*. 2022;11(5):317-326. doi:10.1302/2046-3758.115.BJR-2021-0445.R1

2. Pm G, Ko C, J R, et al. Update of the Healthy Eating Index: HEI-2010. *Journal of the Academy of Nutrition and Dietetics*. 2013;113(4). doi:10.1016/j.jand.2012.12.016

3. Krebs-Smith SM, Pannucci TE, Subar AF, et al. Update of the Healthy Eating Index: HEI-2015. *J Acad Nutr Diet*. 2018;118(9):1591-1602. doi:10.1016/j.jand.2018.05.021

# Supplementary Tables & Figure

## Supplementary Tables

#### **Table S1. The blood metals concentrations of participants stratified by the blood Se quartile.**

| **Metals (50th [20th, 75th])** | **Overall (n=3,842)** | **Q1 (n=961)** | **Q2 (n=960)** | **Q3 (n=960)** | **Q4 (n=961)** | ***p*-value** |
| --- | --- | --- | --- | --- | --- | --- |
| Se, (ug/L) | 193.01 (177.86, 208.97) | 166.75 (158.13, 173.54) | 185.84 (181.62, 189.12) | 200.35 (196.80, 204.04) | 223.02 (214.86, 235.42) | <0.001 |
| Cd, (ug/L) | 0.38 (0.24, 0.66) | 0.42 (0.26, 0.77) | 0.39 (0.24, 0.66) | 0.34 (0.23, 0.60) | 0.37 (0.23, 0.63) | <0.001 |
| Pb, (ug/L) | 1.37 (0.94, 2.06) | 1.36 (0.92, 2.10) | 1.40 (0.94, 2.04) | 1.38 (0.94, 2.07) | 1.36 (0.95, 2.01) | 0.870 |

#### **Table S2. β (95% confidence intervals) of HGS across quartiles Se.**

| Outcomes |  | Single Metal (unadjusted) | | Single Metal (adjusted)^a^ | | Multiple Metals (adjusted)^b^ | |
| --- | --- | --- | --- | --- | --- | --- | --- |
|  | Se | β(95 % CI) | P value | β(95 % CI) | P value | β(95 % CI) | P value |
| Max HGS |  |  |  |  |  |  |  |
|  | Q1 | Reference |  | Reference |  | Reference |  |
|  | Q2 | 1.63(0.71,2.55) | <0.001 | 1.33(0.72,1.94) | <0.001 | 1.33(0.72, 1.94) | <0.001 |
|  | Q3 | 2.77(1.86,3.69) | <0.001 | 1.13(0.52,1.75) | <0.001 | 1.12(0.51, 1.74) | <0.001 |
|  | Q4 | 3.34(2.42,4.25) | <0.001 | 1.23(0.61,1.85) | <0.001 | 1.20(0.58, 1.82) | <0.001 |
| Combined HGS |  |  |  |  |  |  |  |
|  | Q1 | Reference |  | Reference |  | Reference |  |
|  | Q2 | 3.34(1.50,5.17) | <0.001 | 2.64(1.41,3.87) | <0.001 | 2.67(1.44,3.89) | <0.001 |
|  | Q3 | 5.70(3.86,7.54) | <0.001 | 2.34(1.10,3.57) | <0.001 | 2.36(1.12, 3.60) | <0.001 |
|  | Q4 | 6.74(4.90,5.87) | <0.001 | 2.43(1.19,3.67) | <0.001 | 2.44(1.19, 3.69) | <0.001 |

^a^ Covariates in adjusted single-metal models included age, gender, race, education levels, marital status, cotinine, drinking status, BMI, SBP, DBP, HbA1c, and TC.

^b^ Covariates in multiple-metal models included covariates in adjusted single-metal models and the other three metals except for the independent variable.

#### **Table S3. Stratified analysis of associations between Cd and Pb with HGS according to t****he quartiles of Se.**

| Outcomes | Exposure | Se | β | (95 % CI) | *P-value* |
| --- | --- | --- | --- | --- | --- |
| Max HGS |  |  |  |  |  |
|  | Cd | Q1 | -1.37 | (-2.29, -0.49) | 0.002 |
|  |  | Q2 | -0.39 | (-1.47, 0.68) | 0.471 |
|  |  | Q3 | -0.58 | (-1.36, 0.20) | 0.145 |
|  |  | Q4 | 0.03 | (-0.71, 0.76) | 0.938 |
|  | Pb | Q1 | -0.39 | (-0.65, -0.12) | 0.004 |
|  |  | Q2 | -0.09 | (-0.29, 0.10) | 0.336 |
|  |  | Q3 | -0.29 | (-0.58, -0.02) | 0.035 |
|  |  | Q4 | -0.19 | (-0.47, 0.09) | 0.197 |
| Combined HGS |  |  |  |  |  |
|  | Cd | Q1 | -2.75 | (-4.53, -0.97) | 0.002 |
|  |  | Q2 | -0.94 | (-3.07, 1.19) | 0.386 |
|  |  | Q3 | -1.43 | (-3.01, 0.14) | 0.074 |
|  |  | Q4 | -0.21 | (-1.71, 1.29) | 0.786 |
|  | Pb | Q1 | -0.74 | (-1.27, -0.20) | 0.007 |
|  |  | Q2 | -0.15 | (-0.54, 0.24) | 0.457 |
|  |  | Q3 | -0.53 | (-1.10, 0.03) | 0.064 |
|  |  | Q4 | -0.37 | (-0.95, 0.21) | 0.212 |

Adjusted by age, gender, race, education levels, marital status, cotinine, drinking status, BMI, SBP, DBP, HbA1c, and TC.

#### **Table S4. Associations between Se-Cd exposure, Se-Pb exposure, Se-Hg exposure and HGS using general linear regression.**

| Outcomes | Max HGS | | Combined HGS | |
| --- | --- | --- | --- | --- |
|  | β (95 % CI) | P value | β (95 % CI) | P value |
| Se-Pb exposure |  |  |  |  |
| Se-Pb- | Reference |  | Reference |  |
| Se-Pb+ | -0.95(-1.58, -0.32) | 0.003 | -2.12(-3.38, -0.85) | 0.001 |
| Se+Pb- | 0.12(-0.48, 0.73) | 0.688 | 0.22(-1.01, 1.45) | 0.725 |
| Se+Pb+ | -0.14(-0.77, 0.49) | 0.664 | -0.40(-1.67, 0.87) | 0.538 |
| Se-Cd exposure |  |  |  |  |
| Se-Cd- | Reference |  | Reference |  |
| Se-Cd+ | -0.57(-1.22, 0.07) | 0.082 | -1.16(-2.46, 0.14) | 0.080 |
| Se+Cd- | 0.39(-0.22, 0.99) | 0.212 | 0.86(-0.36, 2.08) | 0.167 |
| Se+Cd+ | -0.04(-0.69, 0.62) | 0.914 | -0.10(-1.42, 1.21) | 0.876 |

Adjusted by age, gender, race, education levels, marital status, cotinine, drinking status, BMI, SBP, DBP, HbA1c, and TC.

**Table S5. Qgcomp regression to assess the association of the mixture of blood metals (Cd, Pb) with hand grip strength across Se quartiles.**

| Outcomes | Se | β | (95 % CI) | P value |
| --- | --- | --- | --- | --- |
| Max HGS |  |  |  |  |
|  | Total population | -0.68 | (-0.96, -0.40) | <0.001 |
|  | Q1 | -1.10 | (-1.71, -0.49) | <0.001 |
|  | Q2 | -0.62 | (-1.19, -0.06) | 0.031 |
|  | Q3 | -0.85 | (-1.34, -0.29) | 0.003 |
|  | Q4 | -0.18 | (-0.71, 0.34) | 0.496 |
| Combined HGS |  |  |  |  |
|  | Total population | -1.51 | (-2.07, -0.95) | <0.001 |
|  | Q1 | -2.50 | (-3.72, -1.28) | 0.004 |
|  | Q2 | -1.29 | (-2.42, -0.17) | 0.024 |
|  | Q3 | -1.76 | (-2.88, -0.64) | 0.003 |
|  | Q4 | -0.49 | (-1.56, 0.58) | 0.366 |

Adjusted by age, gender, race, education levels, marital status, cotinine, drinking status, BMI, SBP, DBP, HbA1c, and TC.

#### **Table S6. Qgcomp regression to assess the association of the mixture of blood metals (Se, Cd and Pb) with hand grip strength.**

| Outcomes | Exposure | β | 95%CI | *p*-value |
| --- | --- | --- | --- | --- |
| Max HGS |  |  |  |  |
|  | Se+Pb | -0.25 | (-0.82, 0.33) | 0.401 |
|  | Se+Cd | -0.21 | (-0.81, 0.39) | 0.500 |
|  | Se+Pb+Cd | -0.83 | (-1.53, -0.14) | 0.017 |
| Combined HGS |  |  |  |  |
|  | Se+Pb | -0.04 | (-0.03, 0.24) | 0.755 |
|  | Se+Cd | -0.01 | (-0.31, 0.29) | 0.946 |
|  | Se+Pb+Cd | -0.34 | (-0.68, 0.00) | 0.052 |

Adjusted by age, gender, race, education levels, marital status, cotinine, drinking status, BMI, SBP, DBP, HbA1c, and TC.

#### **Table S7. Qgcomp regression to assess the association of the mixture of blood metals (Cd, Pb) with HGS across Se quartiles. (grouped by gender)**

| Outcomes | Male | | | | Female | | |
| --- | --- | --- | --- | --- | --- | --- | --- |
|  | Se | β | (95 % CI) | P value | β | (95 % CI) | P value |
| Max HGS |  |  |  |  |  |  |  |
|  | Total population | -0.29 | (-0.75, -0.16) | 0.199 | -0.64 | (-0.95, -0.32) | <0.001 |
|  | Q1 | -0.93 | (-1.99, 0.14) | 0.089 | -0.67 | (-1.33, -0.01) | 0.045 |
|  | Q2 | -0.31 | (-1.27, 0.64) | 0.524 | -0.53 | (-1.13, 0.08) | 0.087 |
|  | Q3 | -0.69 | (-1.56, 0.19) | 0.126 | -0.65 | (-1.28, -0.02) | 0.044 |
|  | Q4 | 0.07 | (-0.76, 0.91) | 0.862 | -0.77 | (-1.41, -0.13) | 0.019 |
| Combined HGS |  |  |  |  |  |  |  |
|  | Total population | -0.85 | (-1.78, 0.07) | 0.069 | -1.34 | (-1.97, -0.72) | <0.001 |
|  | Q1 | -1.99 | (-4.14, 0.15) | 0.069 | -1.52 | (-2.85, -0.20) | 0.024 |
|  | Q2 | -0.93 | (-2.84, 0.97) | 0.337 | -0.95 | (-2.14, 0.23) | 0.116 |
|  | Q3 | -1.16 | (-2.94, 0.62) | 0.202 | -1.44 | (-2.68, -0.19) | 0.024 |
|  | Q4 | -0.28 | (-1.97, 1.40) | 0.742 | -1.49 | (-2.82, -0.16) | 0.028 |

Adjusted by age, gender, race, education levels, marital status, cotinine, drinking status, BMI, SBP, DBP, HbA1c, TC, MET score and HEI-2015 index.

#### **Table S8. Qgcomp regression to assess the association of the mixture of blood metals (Cd, Pb) with HGS across Se quartiles. (grouped by age)**

| Outcomes | <60 years old | | | | ≥60years old | | |
| --- | --- | --- | --- | --- | --- | --- | --- |
|  | Se | β | (95 % CI) | P value | β | (95 % CI) | P value |
| Max grip strength |  |  |  |  |  |  |  |
|  | Total population | -0.47 | (-0.91, -0.04) | 0.036 | -0.56 | (-0.94, -0.19) | 0.003 |
|  | Q1 | -1.03 | (-2.16, 0.09) | 0.074 | -0.86 | (-1.63, -0.09) | 0.028 |
|  | Q2 | -1.24 | (-2.11, -0.37) | 0.005 | 0.08 | (-0.67, 0.83) | 0.838 |
|  | Q3 | -0.64 | (-1.52, 0.24) | 0.156 | -0.65 | (-1.37, 0.08) | 0.081 |
|  | Q4 | 0.31 | (-0.49, 1.12) | 0.451 | -0.62 | (-1.34, 0.14) | 0.110 |
| Combined grip strength |  |  |  |  |  |  |  |
|  | Total population | -0.98 | (-1.86, -0.09) | 0.030 | -1.33 | (-2.08, -0.58) | <0.001 |
|  | Q1 | -2.53 | (-4.84, -0.22) | 0.033 | -1.95 | (-3.49, -0.42) | 0.013 |
|  | Q2 | -2.21 | (-3.93, -0.49) | 0.012 | -0.12 | (-1.62, 1.38) | 0.874 |
|  | Q3 | -1.15 | (-2.91, 0.59) | 0.197 | -1.45 | (-2.93, 0.03) | 0.056 |
|  | Q4 | 0.39 | (-1.26, 2.06) | 0.637 | -1.45 | (-2.97, 0.07) | 0.063 |

Adjusted by age, gender, race, education levels, marital status, cotinine, drinking status, BMI, SBP, DBP, HbA1c, TC, MET score and HEI-2015 index.

#### **Table S9. Qgcomp regression to assess the association of the mixture of blood metals (Cd, Pb) with HGS across Se quartiles. (grouped by diabetes)**

| Outcomes | Potential diabetes | | | | No diabetes | | |
| --- | --- | --- | --- | --- | --- | --- | --- |
|  | Se | β | (95 % CI) | P value | β | (95 % CI) | P value |
| Max grip strength |  |  |  |  |  |  |  |
|  | Total population | -0.62 | (-1.31, 0.08) | 0.082 | -0.53 | (-0.84, -0.22) | < 0.001 |
|  | Q1 | -2.12 | (-3.85, -0.39) | 0.018 | -0.92 | (-1.59, -0.26) | 0.006 |
|  | Q2 | 0.12 | (-1.26, 1.49) | 0.868 | -0.57 | (-1.21, 0.05) | 0.074 |
|  | Q3 | -0.78 | (-2.48, 0.92) | 0.369 | -0.76 | (-1.36. -0.17) | 0.011 |
|  | Q4 | -0.69 | (-1.95, 0.57) | 0.282 | 0.04 | (-0.58, 0.66) | 0.897 |
| Combined grip strength |  |  |  |  |  |  |  |
|  | Total population | -1.48 | (-2.89, -0.07) | 0.039 | -1.19 | (-1.82, -0.56) | < 0.001 |
|  | Q1 | -4.60 | (-8.23, -0.97) | 0.014 | -2.11 | (-3.44, -0.78) | 0.001 |
|  | Q2 | 0.16 | (-2.51, 2.83) | 0.907 | -1.15 | (-2.41, 0.10) | 0.073 |
|  | Q3 | -2.07 | (-5.51, -1.18) | 0.239 | -1.48 | (-2.68, -0.28) | 0.016 |
|  | Q4 | -1.78 | (-4.38, 0.83) | 0.184 | -0.09 | (-1.34, 1.16) | 0.888 |

Adjusted by age, gender, race, education levels, marital status, cotinine, drinking status, BMI, SBP, DBP, TC, MET score and HEI-2015 ind

#### **Table S10. Stratified analysis of associations between Cd, Pb, Hg and HGS according to the quartiles of Se after additionally adjusting variables regarding HGS.**

| Outcomes | Exposure | Se | β | (95 % CI) | P value |
| --- | --- | --- | --- | --- | --- |
| Max HGS |  |  |  |  |  |
|  | Cd | Q1 | -1.28 | (-2.18, -3.86) | 0.005 |
|  |  | Q2 | -0.37 | (-1.45, 0.69) | 0.492 |
|  |  | Q3 | -0.56 | (-1.35, 0.23) | 0.164 |
|  |  | Q4 | 0.19 | (-0.57, 0.95) | 0.627 |
|  | Pb | Q1 | -0.41 | (-0.68, -1.41) | 0.002 |
|  |  | Q2 | -0.09 | (-0.28, 0.11) | 0.373 |
|  |  | Q3 | -0.31 | (-0.59, -0.03) | 0.032 |
|  |  | Q4 | -0.13 | (-0.47, 0.21) | 0.453 |
| Combined HGS |  |  |  |  |  |
|  | Cd | Q1 | -2.51 | (-4.32, -0.70) | 0.006 |
|  |  | Q2 | -0.88 | (-3.01, 1.24) | 0.414 |
|  |  | Q3 | -1.44 | (-3.04, 0.15) | 0.076 |
|  |  | Q4 | 0.08 | (-1.46, 1.62) | 0.919 |
|  | Pb | Q1 | -0.76 | (-1.31, -2.22) | 0.005 |
|  |  | Q2 | -0.13 | (-0.51, 0.26) | 0.516 |
|  |  | Q3 | -0.56 | (-1.13, 0.11) | 0.054 |
|  |  | Q4 | -0.29 | (-0.98, 0.40) | 0.404 |

Adjusted by age, gender, race, education levels, marital status, cotinine, drinking status, BMI, SBP, DBP, HbA1c, TC, MET score and HEI-2015 index.

#### **Table S11. Qgcomp regression to assess the association of the mixture of blood metals (Cd, Pb) with HGS across Se quartiles after additionally adjusting variables regarding HGS.**

| Outcomes | Se | β | (95 % CI) | P value |
| --- | --- | --- | --- | --- |
| Max HGS |  |  |  |  |
|  | Total population | -0.62 | (-0.90, -0.33) | <0.001 |
|  | Q1 | -0.97 | (-1.60, -0.35) | 0.002 |
|  | Q2 | -0.61 | (-1.18, -0.03) | 0.037 |
|  | Q3 | -0.86 | (-1.12, -0.29) | 0.003 |
|  | Q4 | -0.11 | (-0.65, 0.43) | 0.689 |
| Combined HGS |  |  |  |  |
|  | Total population | -1.39 | (-1.96, -0.82) | <0.001 |
|  | Q1 | -2.25 | (-3.51, -0.98) | <0.001 |
|  | Q2 | -1.23 | (-2.37, -0.10) | 0.033 |
|  | Q3 | -1.77 | (-2.91, -0.64) | 0.002 |
|  | Q4 | -0.39 | (-1.50, 0.70) | 0.479 |

Adjusted by age, gender, race, education levels, marital status, cotinine, drinking status, BMI, SBP, DBP, HbA1c, TC, MET score and HEI-2015 index.

#### **Table S12. The indicators relative to HGS stratified by gender.**

|  | **Male**  **(n = 1,878)** | | | |  | **Female**  **(n = 1,964)** | | | |
| --- | --- | --- | --- | --- | --- | --- | --- | --- | --- |
| HGS  Se | **Q1**  **(n=437)** | **Q2**  **(n=427)** | **Q3**  **(n=483)** | **Q4**  **(n=531)** |  | **Q1**  **(n=524)** | **Q2**  **(n=533)** | **Q3**  **(n=477)** | **Q4**  **(n=430)** |
| Max HGS, (Kg) | 38.8±9.31 | 41.1±8.65 | 41.1±8.33 | 41.1±8.64 |  | 24.9±6.07 | 26.0±5.85 | 26.3±5.87 | 26.6±5.79 |
| Combined HGS, (Kg) | 77.1±19.0 | 82.0±17.3 | 82.0±17.1 | 81.2±17.5 |  | 50.2±12.2 | 52.0+11.6 | 52.5±11.6 | 53.4±11.7 |

#### **Table S13. The indicators relative to HGS stratified by age.**

|  | **< 60 years**  **(n = 1,715)** | | | |  | **≥ 60 years**  **(n = 2,127)** | | | |
| --- | --- | --- | --- | --- | --- | --- | --- | --- | --- |
| HGS  Se | **Q1**  **(n=387)** | **Q2**  **(n=435)** | **Q3**  **(n=430)** | **Q4**  **(n=463)** |  | **Q1**  **(n=574)** | **Q2**  **(n=525)** | **Q3**  **(n=530)** | **Q4**  **(n=498)** |
| Max HGS, (Kg) | 33.3±10.2 | 33.1±10.2 | 36.3±10.1 | 36.1±10.2 |  | 27.2±9.47 | 28.7±9.87 | 29.6±9.62 | 30.3±9.64 |
| Combined HGS, (Kg) | 66.4±20.3 | 66.7±20.3 | 71.6±20.3 | 71.7±20.6 |  | 53.7±18.8 | 56.7±19.6 | 58.5±19.4 | 60.8±19.1 |

#### Table S14.The Qgcomp analysis with right and left hand serving as the outcome indicators.

| Outcomes | Male | | | | Female | | |
| --- | --- | --- | --- | --- | --- | --- | --- |
|  | Se | β | (95 % CI) | P value | β | (95 % CI) | P value |
| Right Max HGS |  |  |  |  |  |  |  |
|  | Total population | -0.35 | (-0.83, 0.13) | 0.151 | -0.58 | (-0.92, -0.25) | < 0.001 |
|  | Q1 | -0.84 | (-1.97, 0.29) | 0.147 | -0.67 | (-1.36, 0.02) | 0.058 |
|  | Q2 | -0.23 | (-1.22, 0.76) | 0.645 | -0.51 | (-1.14, 0.12) | 0.116 |
|  | Q3 | -0.66 | (-1.59, 0.28) | 0.171 | -0.60 | (-1.27, 0.07) | 0.081 |
|  | Q4 | -0.26 | (-1.09, 0.58) | 0.551 | -0.56 | (-1.26, 0.14) | 0.117 |
| Left Max HGS |  |  |  |  |  |  |  |
|  | Total population | -0.34 | (-0.81, 0.13) | 0.158 | -0.74 | (-1.06, -0.42) | < 0.001 |
|  | Q1 | -1.01 | (-2.11, 0.09) | 0.072 | -0.85 | (-1.52, -0.18) | 0.013 |
|  | Q2 | -0.64 | (-1.62, 0.33) | 0.198 | -0.46 | (-1.05, 0.13) | 0.130 |
|  | Q3 | -0.54 | (-1.42, 0.34) | 0.230 | -0.76 | (-1.39, -0.13) | 0.018 |
|  | Q4 | 0.23 | (-0.65, 1.11) | 0.609 | -0.92 | (-1.58, -0.24) | 0.007 |

Adjusted by age, gender, race, education levels, marital status, cotinine, drinking status, BMI, SBP, DBP, HbA1c, TC, MET score and HEI-2015 index.

## Supplementary Figure


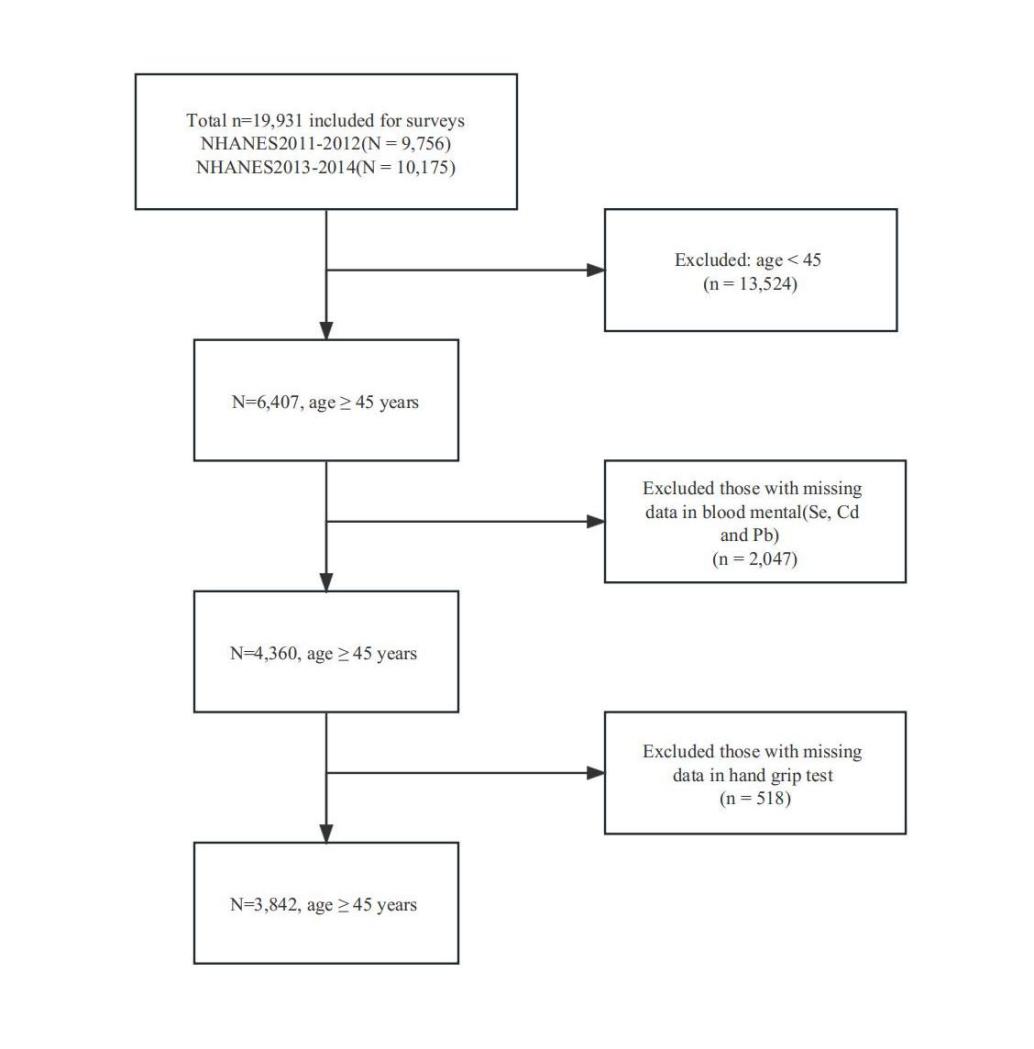


#### **Figure S1** Flow diagram for participants included in the study.
