# Supplementary material for: Moderate selenium mitigates hand grip strength impairment associated with elevated blood cadmium and lead levels in middle-aged and elderly individuals: insights from NHANES 2011–2014
Source: Front Pharmacol. 2023 Dec 14;14:1324583. doi: 10.3389/fphar.2023.1324583 (PMC10757617; doi:10.3389/fphar.2023.1324583)
Supplement: Supplementary file 2 [file Table2.DOCX]

# Moderate selenium alleviates the hand grip strength impairment induced by cadmium and lead in middle-aged and elderly individuals: evidence from HNANES 2011-2014

Yafeng LIANG^1†^, Junqi WANG^2†^, Tianyi WANG^3^, Hangyu LI^4^, Chaohui YIN^5^, Jialin LIU^6^, Yulong WEI^7^, Junxing FAN^8^, Shixing FENG^469*^ and Shuangqing ZHAI^10*^

1 Beijing University of Chinese Medicine, Beijing, China.

2 Dongzhimen Hospital, Beijing University of Chinese Medicine, Beijing, China.

3 School of Management, Beijing University of Chinese Medicine, Beijing, China.

4 School of Life and Science, Beijing University of Chinese Medicine, Beijing, China.

5 School of Resources and Environment, Henan Agricultural University, Zhengzhou, Henan, China.

6 Dongfang Hospital, Beijing University of Chinese Medicine, Beijing, China.

7 School of Acupuncture-Moxibustion and Tuina, Beijing University of Chinese Medicine, Beijing, China.

8 Henan Provincial Health Talent Center, Zhengzhou, Henan, China.

9 Centre France Chine de la Médecine Chinoise, Selles sur Cher, France.

10 School of Traditional Chinese Medicine, Beijing University of Chinese Medicine, Beijing, China.

†These authors contributed equally to this work and share first authorship.

*Correspondence author

Shixing Feng, Beijing University of Chinese Medicine, Beijing 100029, China.

Tel.: +86 15201553363.

E-mail address: [fsx@bucm.edu.cn](mailto:fsx@bucm.edu.cn) (Shixing Feng)

Shuangqing ZHAI, School of Traditional Chinese Medicine, Beijing University of Chinese Medicine, Beijing, China.

Tel.: +86 13501362098.

E-mail address: [zsq2098@163.com](mailto:zsq2098@163.com) (Shuangqing ZHAI)

**The authors:**

**1：Yafeng LIANG**

*Email:* doctorliang2011@163.com

*Affiliation*: Beijing University of Chinese Medicine, Beijing, China.

**2：Junqi WANG**

*Email:* coewjq@pku.edu.cn

*Affiliation*: Dongzhimen Hospital, Beijing University of Chinese Medicine, Beijing, China.

**3：Tianyi WANG**

*Email:* w760361263@163.com

*Affiliation*: School of Management, Beijing University of Chinese Medicine, Beijing, China.

**4：Hangyu LI**

*Email:* lhy@bucm.edu.cn

*Affiliation*: School of Life and Science, Beijing University of Chinese Medicine, Beijing, China.

**5：Chaohui YIN**

*Email*: [chaohuiyin@163.com](mailto:chaohuiyin@163.com)

*Affiliation*: School of Resources and Environment, Henan Agricultural University, Zhengzhou, Henan, China.

**6：Jialin LIU**

*Email:* Jordan181@163.com

*Affiliation*: Dongfang Hospital, Beijing University of Chinese Medicine, Beijing, China.

**7：Yulong WEI**

*Email:* wyl_5128@163.com

*Affiliation*: School of Acupuncture-Moxibustion and Tuina, Beijing University of Chinese Medicine, Beijing, China.

**8：Junxing FAN**

*Email:* 2393849000@qq.com

*Affiliation*: Henan Provincial Health Talent Center, Zhengzhou, Henan, China.

**9：Shixing FENG (Corresponding Author)**

*Email*: [fsx@bucm.edu.cn](mailto:fsx@bucm.edu.cn)

*Affiliation*: School of Life and Science, Beijing University of Chinese Medicine, Beijing, China; Dongfang Hospital, Beijing University of Chinese Medicine, Beijing, China; Centre France Chine de la Médecine Chinoise, Selles sur Cher, France.

Contact Information

- Email: [fsx@bucm.edu.cn](mailto:fsx@bucm.edu.cn)

- Phone: +86 15201553363

Postal address: Dongfang Hospital, Beijing University of Chinese Medicine, Beijing, China.

**10：Shuangqing ZHAI (Corresponding Author)**

*Email*: [zsq2098@163.com](mailto:zsq2098@163.com)

*Affiliation*: School of Traditional Chinese Medicine, Beijing University of Chinese Medicine, Beijing, China.

Contact Information

- Email: [zsq2098@163.com](mailto:zsq2098@163.com)

- Phone: +86 13501362098

Postal address: School of Traditional Chinese Medicine, Beijing University of Chinese Medicine, Beijing, China.

**Word count**

5035 words.(excluding abstract, research in context, references, acknowledgements, funding statement, authors’ relationships and activities, contribution statement, tables and figure legends)
